# Transcriptomic Analysis of Responses to Imbalanced Carbon: Nitrogen Availabilities in Rice Seedlings

**DOI:** 10.1371/journal.pone.0165732

**Published:** 2016-11-07

**Authors:** Aobo Huang, Yuying Sang, Wenfeng Sun, Ying Fu, Zhenbiao Yang

**Affiliations:** 1 State Key Laboratory of Plant Physiology and Biochemistry, College of Biological Sciences, China Agricultural University, Beijing, China; 2 Haixia Institute of Science and Technology, Horticultural Plant Biology and Metabolomics Center, Fujian Agriculture and Forestry University, Fuzhou, China; 3 National Key Laboratory of Plant Molecular Genetics, Institute of Plant Physiology and Ecology, Shanghai Institute for Biological Sciences, Chinese Academy of Sciences, Shanghai, China; 4 Center for Plant Cell Biology, Institute of Integrated Genome Biology, and Department of Botany and Plant Sciences, University of California Riverside, Riverside, California, United States of America; Hainan University, CHINA

## Abstract

The internal C:N balance must be tightly controlled for the normal growth and development of plants. However, the underlying mechanisms, by which plants sense and balance the intracellular C:N status correspondingly to exogenous C:N availabilities remain elusive. In this study, we use comparative gene expression analysis to identify genes that are responsive to imbalanced C:N treatments in the aerial parts of rice seedlings. Transcripts of rice seedlings treated with four C:N availabilities (1:1, 1:60, 60:1 and 60:60) were compared and two groups of genes were classified: high C:low N responsive genes and low C:high N responsive genes. Our analysis identified several functional correlated genes including *chalcone synthase* (*CHS*), *chlorophyll a-b binding protein* (*CAB*) and other genes that are implicated in C:N balancing mechanism, such as *alternative oxidase 1B* (*OsAOX1B*), *malate dehydrogenase* (*OsMDH*) and *lysine and histidine specific transporter 1* (*OsLHT1*). Additionally, six jasmonate synthetic genes and key regulatory genes involved in abiotic and biotic stresses, such as *OsMYB4*, *autoinhibited calcium ATPase 3* (*OsACA3*) and *pleiotropic drug resistance 9* (*OsPDR9*), were differentially expressed under high C:low N treatment. Gene ontology analysis showed that high C:low N up-regulated genes were primarily enriched in fatty acid biosynthesis and defense responses. Coexpression network analysis of these genes identified eight *jasmonate ZIM domain protein* (*OsJAZ*) genes and several defense response related regulators, suggesting that high C:low N status may act as a stress condition, which induces defense responses mediated by jasmonate signaling pathway. Our transcriptome analysis shed new light on the C:N balancing mechanisms and revealed several important regulators of C:N status in rice seedlings.

## Introduction

Carbon (C) and nitrogen (N) balance is universal and critical for metabolism, growth and development in all cellular organisms. To cope with changing environment conditions, cellular organisms have to sense the nutrient supply and adjust their metabolism accordingly. In cyanobacteria and *Escherichia coli*, a highly conserved protein, named PII, has been known to act as a key regulator for integrating C and N metabolism by sensing α-ketoglutarate and adenylate energy charge [1, 2]. In yeast and mammals, metabolism of C and N are mainly integrated by regulating processes such as the tricarboxylic acid (TCA) cycle and N assimilation through the crosstalk between pathways involving the target of rapamycin (TOR) and sucrose nonfermenting 1 (SNF1) kinases/AMP-activated kinases (AMPK) [3–5]. Similarly, the interaction between C and N metabolic pathway is indispensible for normal plant growth and development. CO_2_ fixation and assimilation into photosynthetic products, mainly sucrose and glucose, is dependent on N uptake and assimilation, particularly because a large number of proteins and enzymes (N products), such as RuBisCo (Ribulose-1,5-bisphosphate carboxylase/oxygenase) and thylakoid proteins, are required in large quantities for the photosynthetic processes. Conversely, photosynthetic products provide C skeletons and energy essential for the incorporation of inorganic N into amino acids and other biologically important molecules. Therefore, C and N metabolisms need to be tightly coordinated to maintain a balance between C and N metabolites in plants [6–8]. For example, when plants are supplied with limited N, carbohydrates accumulate in leaves, which would result in the repression of photosynthetic genes [9–11]. On the other hand, when plants are supplied with sufficient N, most N is allocated into chloroplasts but not mitochondria, and this is reflected in differences of photosynthesis and respiration rates with the final results of the accumulation of carbohydrates [12].

Despite mechanisms for C:N balancing are well studied in unicellular organisms and mammals, they are still elusive in plants. This is in part due to the complexity of C:N balancing in plants, as C and N are up-taken through aerial parts and roots, respectively, and need to be redistributed dynamically among different organs [13, 14]. However, understanding the C:N balancing mechanism in plants is of great significance because of their major impacts on crop yield. One way to maintain a proper C:N balance in plants is to regulate the growth of roots and leaves, which uptake and fix N and C, respectively. For example, high nitrate levels inhibit lateral root growth to limit N uptake in *Arabidopsis* [15, 16]. Similarly, high levels of sucrose or glucose inhibit the shoot growth of plants [17, 18]. Recently, a bZIP transcription factor elongated hypocotyls 5 (HY5), which acts as a shoot-to-root mobile signal that mediates C assimilation in the shoot and light promotion of root growth, is suggested to contribute greatly to the maintenance of homeostatic C and N balance in *Arabidopsis* [19]. Apart from the regulation of C:N balance by N and C status, evidence supports a role for C:N ratio or imbalance in this regulation [13, 20, 21]. For example, *nitrate reductase* (*NR*) could be induced by nitrate [22], which is alleviated, however, when cellular carbohydrate content is limited [23]. *Chalcone synthase* (*CHS*) is up-regulated by sugar treatment [24], but under low N condition only [25]. These initial findings raised an interesting question: Do plants have a mechanism for sensing C:N ratios or imbalance? Alternatively, C and N sensors could cross-talk with each other to regulate C:N balance. Microarray analysis revealed over 300 genes were differentially expressed in *Arabidopsis* seedlings subjected to combined C:N treatments compared to C or N treatments respectively, supporting the hypothesis that plants have a C:N sensing mechanism [7]. Besides, studies using *Arabidopsis* seedlings treated with different C:N availabilities (0:60, 0:0.1, 100:60, and 100:0.1) suggested that C:N ratio but not C and N alone played major roles in regulating plant growth [26]. However, another group, in their searching of global gene expression patterns to a matrix of C:N treatments, failed to identify any C:N ratio responsive genes and suggested that the C:N ratio was not a predominant regulatory mechanism in plants [6]. The different conclusions of these studies implied the difficulty in revealing the molecular mechanisms underlying the C:N balance in plants.

Several genetic studies support the capability of plants to sense C:N imbalance. *Glutamate receptor homolog 1*.*1* (*GLR1*.*1*), encoding a putative glutamate receptor, is necessary in seed germination under the exogenous high C:low N condition [27]. *Oversensitive to sugar 1* (*OSU1*), encoding a putative methyltransferase, is indispensible for the adaptation to imbalanced C:N conditions [28]. *Carbon/nitrogen insensitive 1* (*CNI1*)*/Arabidopsis toxicos en levadura 31* (*ATL31*), a RING-type ubiquitin ligase encoding gene, functions in growth phase transition and leaf senescence under high C:low N condition [29, 30]. Imbalanced C:N is also found to regulate the expression of genes that are involved in C and N metabolism in rice [18]. These findings prompted us to speculate that rice plants may sense imbalanced C:N conditions, either high C:low N or low C:high N, and this sensing may lead to the regulation of C and N metabolism in order to achieve the C:N balance. As a first step to test this notion, we need to identify gene markers for imbalanced C:N.

Rice is an important model for cereals and a staple crop worldwide. Understanding C-N nutrients interaction in rice should also help to compare and contrast the mechanisms for the regulation of C:N balance between plants accumulating high levels of photosynthates such as cereals and those that do not, and importantly may provide useful knowledge for improving crop yields. By analyzing the effects of exogenous C:N availabilities on the growth of rice seedlings, we previously identified the balanced and imbalanced C:N conditions for rice seedlings [18]. Herein, we chose four C:N ratios (1 mM:1 mM, 1 mM:60 mM, 60 mM:1 mM and 60 mM:60 mM). Among them, 1:1 and 60:60 were considered as balanced exogenous C:N while 60:1 and 1:60 were imbalanced C:N. By comparative transcriptome analysis, genes were classified into two modes: high C:low N responsive genes and low C:high N responsive genes. A number of C and N regulated genes were identified as the potential regulators of C:N balance. Additionally, several jasmonate signaling and defense response related genes were revealed in high C:low N condition, suggesting that jasmonate signaling might be involved in C:N balancing mechanism under high C:low N conditions.

## Materials and Methods

### Plant materials and growth conditions

Rice seeds (*Oryza sativa* L. ssp. *Japonica* var. Nipponbare) were decorticated and sterilized in 75% (v/v) ethanol for 1 min and then in 20% (v/v) sodium hypochlorite for 20 min. After washing by sterile water for five times, seeds were kept at 37°C for 24 h to accelerate the germination. Then seeds were transferred to the greenhouse (28°C, 14 h light and 10 h dark) for an additional 48 h. Germinated seeds were cultured in 1/3B5 liquid culture medium [31] for three weeks with medium renewal of every 2 days. Then rice seedlings were refreshed with culture medium without N nutrients for 48 h before the treatments. Four C:N availabilities (1 mM C:1 mM N, 1 mM C:60 mM N, 60 mM C:1 mM N, 60 mM C:60 mM N) were used for the treatments. Sucrose was used as the C source, and the combination of NO_3_^-^ and NH_4_^+^ in a molar ratio of 12:1 was used as the N source. All of the media contained similar amount of K^+^ by replacing KNO_3_ with KCl, if necessary. Aerial parts of rice seedlings were harvested at 0, 1, 2, 3 and 4 h after the treatments and kept at -80°C until further analysis.

### Purification of total RNA

Total RNA was extracted by RNeasy Plant Mini Kit (Qiagen, Hilden, Germany) according to the manufacturer’s instructions, including a DNase digestion step. The yield and RNA quality were assessed using a spectro-photometer NanoDrop 2000 (NanoDrop Technologies, Wilmington, DE, USA) and a Bioanalyzer 2100 (Agilent Technologies, Santa Clara, CA, USA).

### Real-time quantitative PCR analysis

The first-strand cDNA was synthesized by M-MLV reverse transcriptase (Promega, Madison, WI, USA) from 2 μg total RNA. SYBP Green Premix Ex Taq^TM^ II (Takara-Bio, Otsu, Shiga, Japan) and the ABI StepOne Plus^TM^ Real-Time PCR system (Applied Biosystems, Foster City, CA, USA) were used for the detection. Running conditions were 95°C for 30 s, 40 cycles of 95°C for 5 s, 60°C for 15 s, and 72°C for 20 s. Then melting curves were performed to detect the specificities of primers. *Actin6* (*Os04g0667700*) was used as the internal reference and its primer sequences were 5’-TCAAGCATGTTTACACCCGTCGAG-3’ (forward primer) and 5’-AGCAGCATACTTGCTCCATCCGTA-3’ (reverse primer). The gene-specific primers are listed in S1 Table. The relative quantification method (using the formula: 2^-ΔΔCt^) was used to evaluate quantitative variation between the replicates examined.

### Microarray hybridization

The microarray hybridization was performed by Kangcheng Bio-tech Inc (Shanghai, China). Briefly, total RNA from each sample was amplified and labeled with Cy3-UTP. 1 μg of each labeled cRNA was fragmented with fragmentation buffer (Agilent Technologies) at 60°C for 30 min. The fragmented labeled cRNA was then pooled and hybridized to the Rice Gene Expression Microarray (G2519F-015241; Agilent Technologies) at 60°C for 17 h. After washing and blow-drying with nitrogen gun, microarrays were scanned by Agilent microarray scanner (Agilent Technologies). Scanned images were analyzed by Feature Extraction software (version 11.0.1.1; Agilent Technologies) and the raw gene expression data were imported into GeneSpring GX software package (version 11.5; Agilent Technologies) for further analysis. The 12 microarray data sets were normalized in GeneSpring GX using the Agilent Feature Extraction one-color scenario (mainly using median normalization). All of the data were interpreted using the log-ratio setting. The normalized expression data are listed in S1 File. Microarray raw data are available in the ArrayExpress database (www.ebi.ac.uk/arrayexpress) under accession number E-MTAB-5118.

### Data analysis

Of the total 43,803 probe sets in Rice Gene Expression Microarray, 33,187 probe sets showed signals in all of the twelve samples (each of four C:N treatments contains three biological replicates). Differentially expressed genes were identified by determining the fold-change (FC) and *p*-value of the *t*-test algorithm between two groups (60:1 compared with 1:1, 1:60 compared with 1:1, 60:60 compared with 1:60, 60:60 compared with 60:1) (S2 File). Genes with an FC of ≥ 1.5 and a *p*-value of ≤ 0.05 between two groups were identified as differentially expressed genes. Hierarchical cluster analysis was performed by R software (http://www.r-project.org). AgriGO [32] was used to perform the gene ontology (GO) analysis for functional categorization of high C:low N and low C:high N responsive genes compared with the genome-wide background with an adjusted *p*-value (False discovery rate, FDR) cutoff of 0.05. The GO categories were derived from Gene Ontology (www.geneontology.org) and comprised three structured networks (biological process, cellular component and molecular function) of defined terms to describe the gene product attributes. High C:low N up-regulated genes were chosen to perform coexpression analysis by RiceFREND online web tool [33]. Functional analysis of the coexpression genes was performed using the KEGG pathway database (www.genome.jp/kegg/pathway.html).

## Results

### Experimental design to identify genes responsive to imbalanced C:N

**Fig 1:**
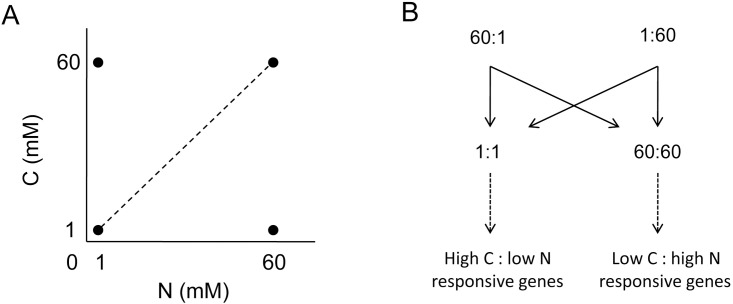
Experimental design to identify genes responsive to imbalanced C:N. (A) N starved rice seedlings were treated with four different C:N conditions: balanced C:N (1:1 and 60:60) or imbalanced C:N (1:60 and 60:1). (B) Hypothetical models of genes responsive to exogenous imbalanced C:N conditions. Genes responsive to imbalanced high C:low N (60:1) or low C:high N (1:60) are proposed to show higher or lower expression levels compared with 1:1 and 60:60 treatments.

To identify genes responsive to imbalanced C:N, we compared gene expression profiles in rice seedlings treated with four C:N availabilities (1:1, 1:60, 60:1, 60:60).These four C:N conditions were chosen based on our previous studies [18]. In brief, 1:1 (1 mM C:1mM N) and 60:60 represent balanced low C:low N and high C:high N, respectively, whereas 1:60 and 60:1 represent imbalanced low C:high N and high C:low N, respectively. Rice seedlings were grown hydroponically in light/dark cycles (14/10 h) for three weeks with 1/3B5 medium (without exogenous C). After nitrogen starvation with nitrogen-free 1/3B5 medium for 48 h, rice seedlings were treated with four C:N conditions and then shoot tissues were collected for RNA expression analysis (Fig 1A). RNA expression profiling was conducted using the Rice Gene Expression Microarray from Agilent (Agilent Technologies). Genes responsive to imbalanced C:N (1:60 and 60:1) would have higher or lower expressions than that under balanced conditions (1:1 and 60:60) (Fig 1B).

### Determination of C:N treatment time for the identification of C:N imbalanced genes

**Fig 2:**
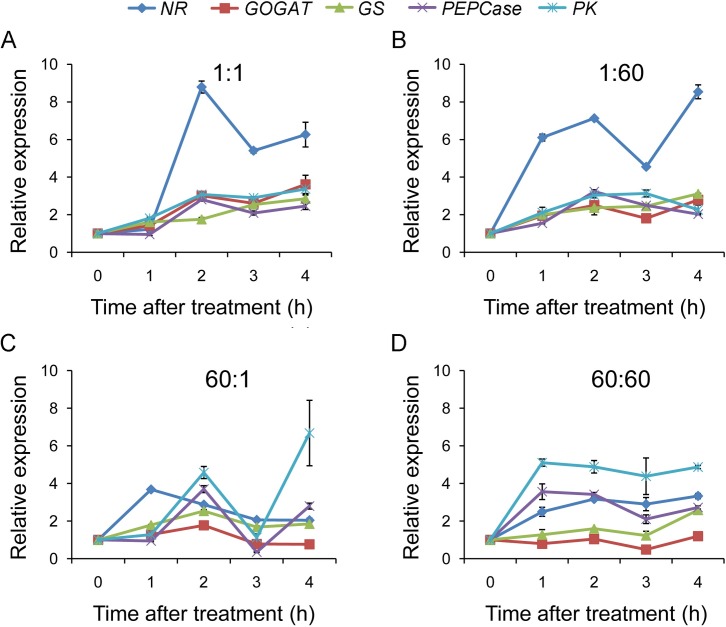
qRT-PCR analysis of CN metabolic genes at different time points after C:N treatments. Expression patterns of *NR*, *GOGAT*, *GS*, *PEPCase* and *PK* were analyzed in rice seedlings treated with four different C:N conditions (A 1:1; B 1:60; C 60:1; D 60:60) for 1, 2, 3 and 4 h. The beginning of the treatment (0 h) was used as the control and *Actin6* served as the internal reference. Values are shown as means ± SDs from three technical replicates. A representative experiment of two biological replicates is shown.

To investigate gene responses of rice seedlings to C:N availabilities, we need to determine an appropriate time duration for the treatments. We investigated time course of responses for several key genes known to be affected by both C and N availabilities including *nitrate reductase* (*NR*), *glutamine oxoglutarate aminotransferase* (*GOGAT*), *glutamine synthase* (*GS*), *phosphoenolpyruvate carboxylase* (*PEPCase*) and *pyruvated kinase* (*PK*) [34]. qRT-PCR analysis showed that treatments with imbalanced C:N (both 1:60 and 60:1) induced transient increases in transcript levels for all genes examined with peaks around 2 h for most of these genes (Fig 2B and 2C). Interestingly, 1:60 treatments tended to cause transcript levels for N assimilatory genes (*NR*, *GS*, *GOGAT*) to increase again after the first peak, while 60:1 treatments did so for C metabolic genes (*PK* and *PEPCase*). Treatments with balanced C:N also induced changes in transcript levels for the C and N metabolic genes, but the patterns of changes were different (Fig 2A and 2D). 1:1 treatments caused slow but steady increases in transcript levels for all genes except for *NR*, which exhibited a transient increase. 60:60 treatments caused rapid and sustained increases in transcript levels for *PK*, *PEPCase*, and *NR*, but seemed to have little effect on *GS* and *GOGAT*. These expression patterns of the C and N metabolic genes not only confirm the importance of C-N interactions in the regulation of their expression as previously described [18, 35], but also reveal unique gene expression pattern under imbalanced C:N conditions. Importantly, these results suggest that treatments with imbalanced C:N for 2 h will most likely uncover C:N imbalance marker genes.

### Identification of high C:low N and low C:high N responsive genes

**Fig 3:**
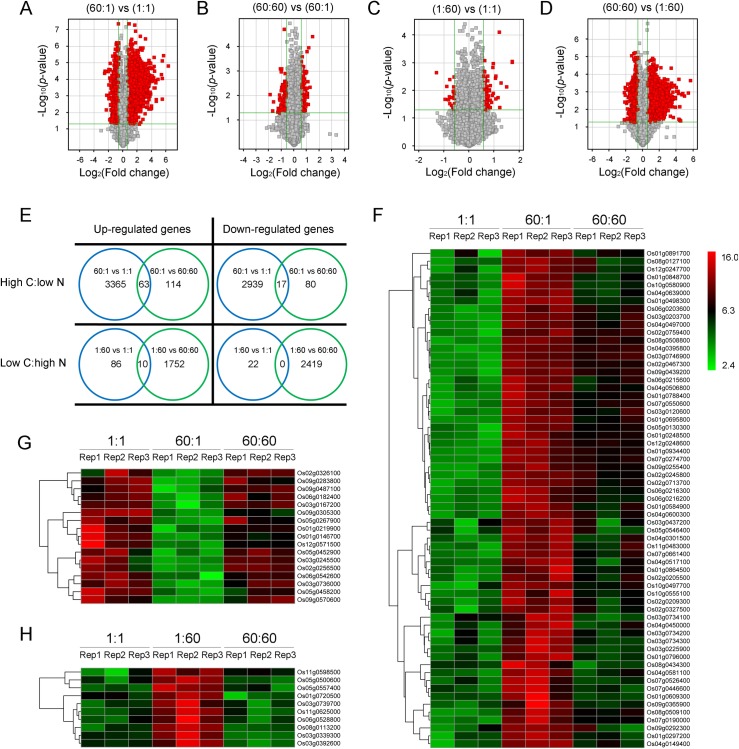
Identification of genes responsive to imbalanced high C:low N and low C:high N. (A-D) The Volcano Plots for differentially expressed genes between treatments. The two vertical lines are the 1.5-fold change boundaries and the horizontal lines are the statistical significance boundaries (*p*<0.05). Genes with fold change>1.5 and statistical significance are marked with red dots. A, 60:1 compared with 1:1; B, 60:60 compared with 60:1; C, 1:60 compared with 1:1; D, 60:60 compared with 1:60. (E) Venn diagram of rice genes (probe sets) responded to C:N treatments. (F-H) Hierarchical cluster analysis of high C:low N and low C:high N responsive genes. The log_2_ ratio values of probe sets were used for the analysis with R software. The colored bars represent the value (log_2_(fold change)) of the transcripts in each bin after C:N treatments. Green represents down-regulated probe sets, red represents up-regulated probe sets, and dark indicates no significant difference in gene expression. F, High C:low N up-regulated genes; G, High C:low N down-regulated genes; H, Low C:high N up-regulated genes. “vs” represents “compared with”.

To identify imbalanced C:N responsive genes, transcriptomic profiles from 60:1 and 1:60 treatments were first compared with those from 1:1 and 60:60 treatments, respectively. Hierarchical clustering was used to evaluate the global differences between treatments and dendrogram representations based on their global genome responses showed the relationships among the treatments (S1 Fig). As the volcano plots shown in Fig 3, large differences were observed in 60:1 vs 1:1 and 60:60 vs 1:60 groups, while relatively small differences were observed in 60:60 vs 60:1 and 1:60 vs 1:1 groups. These results indicated that C, rather than N, has large effect on the expression of genes as previously reported [6, 7]. Besides, more genes were up-regulated than down-regulated by C no matter the concentrations of N source (Fig 3A and 3D). Similarly, there were slightly more genes up-regulated than down-regulated by N under either high C or low C conditions (Fig 3B and 3C).

Based on the above analysis, high C:low N and low C:high N responsive genes could be obtained by identifying the intersections between groups. As the Venn diagram showed (Fig 3E), 63 and 17 genes were categorized as those up- and down-regulated by the high C:low N imbalance, respectively, whereas 10 genes were categorized as those up-regulated by low C:high N imbalance. However, no genes were found to be down-regulated under low C:high N conditions. To gain an insight into the effects of high C:low N and low C:high N status on transcriptomic profiles, we illustrated the expression patterns using heatmaps obtained via hierarchical cluster analysis. Fig 3F and 3G showed the up-regulated and down-regulated genes by high C:low N, respectively. The up-regulated genes by low C:high N were shown in Fig 3H. The annotations of genes listed in the right sides of the heatmaps were described in Table 1.

**Table 1:** Annotations of genes responsive to imbalanced high C:low N and low C:high N.

	Description	Transcription factor	Name
**High C:low N Up**			
*Os01g0891700*	Leucine rich repeat, N-terminal domain containing protein.		
*Os08g0127100*	Lysine and histidine specific transporter.		*OsLHT1*
*Os12g0247700*	Jacalin-like lectin domain containing protein.		*OsJAC1*
*Os01g0848700*	Ras-related protein Rab11C.		
*Os10g0580900*	Conserved hypothetical protein.		
*Os04g0639000*	Conserved hypothetical protein.		
*Os01g0498300*	Protein of unknown function DUF563 family protein.		
*Os06g0203600*	Conserved hypothetical protein.		
*Os03g0203700*	Plasma membrane Ca^2+^-ATPase.		*OsACA3*
*Os04g0497000*	Allyl alcohol dehydrogenase.		
*Os02g0759400*	Zn-finger, RING domain containing protein.		
*Os08g0508800*	Lipoxygenase, chloroplast precursor (EC 1.13.11.12).		*OsLOX*
*Os04g0395800*	ZIM domain containing protein.		*OsJAZ5*
*Os03g0746900*	Protein of unknown function DUF1677 family protein		
*Os02g0467300*	Conserved hypothetical protein.		
*Os09g0439200*	ZIM domain containing protein.		*OsJAZ8*
*Os06g0215600*	Oxo-phytodienoic acid reductase.		*OsOPR5*
*Os04g0506800*	Sialyltransferase.		
*Os01g0788400*	Pectinesterase (EC 3.1.1.11) (Pectin methylesterase).		*OsPME*
*Os07g0550600*	Transferase family protein.		
*Os03g0120600*	Conserved hypothetical protein.		
*Os01g0695800*	Multidrug resistance protein 1 homolog.		
*Os05g0130300*	Conserved hypothetical protein.		
*Os01g0248500*	Pathogen-related protein.		
*Os12g0248600*	Hypothetical protein.		
*Os01g0934400*	Photosystem II complex PsbP family protein.		
*Os07g0274700*	B12D family protein.		
*Os09g0255400*	Indole-3-glycerol phosphate synthase (IGPS).		
*Os02g0245800*	Inwardly rectifying potassium channel.		
*Os02g0713700*	Protein of unknown function DUF296 family protein.		
*Os06g0216300*	Oxo-phytodienoic acid reductase.		*OsOPR1*
*Os06g0216200*	Oxo-phytodienoic acid reductase.		*OsOPR2*
*Os01g0584900*	WRKY transcription factor.	WRKY	*OsWRKY77*
*Os04g0600300*	Alternative oxidase.		*OsAOX1B*
*Os03g0437200*	Zn-finger, C2H2 type domain containing protein.	C2H2	
*Os05g0546400*	Conserved hypothetical protein.		
*Os04g0301500*	Basic helix-loop-helix region containing protein.	bHLH	*OsbHLH6*
*Os11g0483000*	Cytochrome P450 family protein.		
*Os07g0661400*	Conserved hypothetical protein.		
*Os04g0517100*	Myb protein.	MYB	*OsMYB4*
*Os01g0864500*	Harpin-induced 1 domain containing protein.		
*Os02g0205500*	Naringenin-chalcone synthase family protein.		*OsCHS*
*Os10g0497700*	Phytochelatin synthetase.		
*Os10g0555100*	Glucosyltransferase like protein.		
*Os02g0209300*	Non-protein coding transcript, unclassifiable transcript.		
*Os02g0327500*	Protein of unknown function DUF266 family protein.		
*Os03g0734100*	Conserved hypothetical protein.		
*Os04g0450000*	FYVE/PHD zinc finger domain containing protein.		
*Os03g0734200*	Hypothetical protein.		
*Os03g0734300*	Proteinase inhibitor I20, Pin2 family protein.		
*Os03g0225900*	Allene oxide synthase (EC 4.2.1.92).		*OsAOS2*
*Os01g0796000*	(No Hit)		
*Os08g0434300*	Malate dehydrogenase precursor (EC 1.1.1.37).		*OsMDH*
*Os04g0581100*	Flavanone 3-hydroxylase.		
*Os07g0526400*	Chalcone synthase (EC 2.3.1.74).		*OsCHS*
*Os07g0446600*	Hypothetical protein.		
*Os01g0609300*	PDR-like ABC transporter.		*OsPDR9*
*Os09g0365900*	L-ascorbate oxidase precursor (Ascorbase) (ASO).		
*Os08g0509100*	Lipoxygenase, chloroplast precursor (EC 1.13.11.12).		*OsLOX*
*Os07g0190000*	1-deoxy-D-xylulose 5-phosphate synthase 2 precursor.		
*Os09g0292300*	Conserved hypothetical protein.		
*Os01g0297200*	AAA ATPase, central region domain containing protein.		
*Os04g0149400*	Hypothetical protein.		
**High C:low N Down**			
*Os02g0326100*	Non-protein coding transcript, uncharacterized transcript.		
*Os09g0283800*	(No Hit)		
*Os09g0487100*	Hypothetical protein.		
*Os06g0182400*	Metallophosphoesterase domain containing protein.		
*Os03g0167200*	IQ calmodulin-binding region domain containing protein.		
*Os09g0305300*	Plant protein of unknown function family protein.		
*Os05g0267900*	Hypothetical protein.		
*Os01g0219900*	Conserved hypothetical protein.		
*Os01g0146700*	Integrase, catalytic region domain containing protein.		
*Os12g0571500*	Hypothetical protein.		
*Os05g0452900*	Conserved hypothetical protein.		
*Os03g0245500*	Curculin-like lectin domain containing protein.		
*Os02g0256500*	Conserved hypothetical protein.		
*Os06g0542600*	(No Hit)		
*Os03g0736000*	NOT2/NOT3/NOT5 family protein.		
*Os05g0458200*	(No Hit)		
*Os09g0570600*	PAP/25A core domain containing protein.		
**Low C:high N Up**			
*Os11g0598500*	(No Hit)		
*Os05g0500600*	GRAS transcription factor domain containing protein.	GRAS	
*Os05g0557400*	Membrane attack complex component C9 family protein.		
*Os01g0720500*	Chlorophyll a-b binding protein 2 (LHCII type I CAB-2).		*OsCAB2*
*Os03g0739700*	Protein of unknown function DUF1334 family protein.		
*Os11g0625000*	(No Hit)		
*Os06g0528800*	(No Hit)		
*Os08g0113200*	RNA-binding region RNP-1 domain containing protein.		
*Os03g0339300*	Peroxidase (EC 1.11.1.7).		
*Os03g0392600*	Peptidase S10, serine carboxypeptidase family protein.		

Several classes of transcription factors including WRKY, C2H2, bHLH, and MYB were found among the 63 high C:low N up-regulated genes. WRKY proteins comprise a large family of transcription factors in plants, which are involved in various plant processes, especially in coping with diverse biotic and abiotic stresses [36, 37]. The *WRKY* gene (*Os01g0584900*) identified here is classified as *OsWRKY77* [38]. C2H2-type zinc finger protein family, which exists in almost all eukaryotes, constitutes one of the largest families of transcriptional regulators in plants [39]. Recent studies revealed that C2H2 zinc finger proteins function as key transcriptional repressors involved in the defense and acclimation response of plants to different environmental stress conditions [40]. The C2H2 zinc finger protein encoding gene (*Os03g0437200*) identified in this study is reported to be induced in the sheaths of rice seedlings under N-deficiency condition [41]. Basic helix-loop-helix (bHLH) protein family is present throughout the three eukaryotic kingdoms and constitutes one of the largest families of transcription factors [42]. Rice genome contains 167 *bHLH* genes [43], among which *OsbHLH6* (*Os04g0301500*) was found here to be up-regulated by high C:low N. The MYB protein family, which comprises one of the richest groups of transcription factors in plants, is involved in plant development, secondary metabolism, hormone signal transduction, disease resistance, and abiotic stress tolerance [44, 45]. There are more than 155 *MYB* genes in rice genome [46] and *OsMYB4* (*Os04g0517100*) was found to be up-regulated here. Overexpression of *OsMYB4* improves the adaptability of several species to abiotic or biotic stresses, for instance, freezing tolerance in *Arabidopsis* and barley [47, 48], disease tolerance in tomato [49], drought and cold tolerance in apple [50]. Laura et al. found that the improved freezing tolerance of *Osteospermum ecklonis* by overexpressing of *OsMYB4* was companied with the accumulation of sugar and proline, suggesting the involvement of *OsMYB4* in CN metabolism regulation [51].

Several C:N signaling and metabolism related genes were also found in the list of high C:low N up-regulated genes. Lysine and histidine specific transporters (LHTs), which are originally described as lysine and histidine selective transporters [52], can transport a broad spectrum of amino acids, especially neutral and acidic amino acids, in plants [53, 54]. They have been suggested to be involved in the import of organic nitrogen into root and mesophyll cells [55] as well as cells of reproductive floral tissues [53]. Here the identified gene (*Os08g0127100*) encoded one of the six LHTs in rice and was named as *OsLHT1* with high transcripts in root, leaf, inflorescence and seeds [56, 57]. Chalcone synthase (CHS) is a key enzyme in flavonoid biosynthesis pathway, and the expression level of *CHS* is one of the determinants of anthocyanin biosynthesis [58, 59]. *CHS* is induced by the supplement of sugar [24] and the depletion of nitrogen [25, 60] and is considered as a high C:low N marker gene in *Arabidopsis* [26]. Two *CHS* genes (*Os07g0526400* and *Os02g0205500*) were identified to be up-regulated by high C:low N in this study, demonstrating their functional conservation in rice. In addition, a mitochondrial NAD-malate dehydrogenase (MDH) encoding gene (*Os08g0434300*) was identified in our analysis. Plants contain multiple isoforms of malate dehydrogenases that catalyze the interconversion between malate and oxaloacetate coupled to reduction or oxidation of the NAD(P) pool [61]. It was reported that decreased mitochondrial malate dehydrogenase resulted in enhanced photosynthetic performance in tomato [62].

Furthermore, a number of jasmonate signaling genes were identified as high C:low N up-regulated genes. Lipoxygenase (LOX) catalyzes the conversion of polyunsaturated fatty acids into conjugated hydroperoxides [63]. In plants, LOXs are involved in jasmonic acid biosynthesis [64] and response to biotic and abiotic stresses [65]. Two of the fourteen *LOX* genes (*Os08g0508800* and *Os08g0509100*) in rice genome [66] were identified in this study. Allene oxide synthase (AOS) is another key enzyme in jasmonic acid biosynthetic pathway in plants [67], which converts 13(S)-hydroperoxylinolenic acid to the corresponding allene oxide, 12,13-epoxyoctadeca-9,11,15-trienoic acid [68]. The rice genome contains at least five *AOS* genes [68]. The identified one here (*Os03g0225900*) was designated as *OsAOS2* and was reported to be up-regulated by light, heavy metals and pathogen attacks [69, 70]. Oxo-phytodienoic acid reductase (OPR) catalyzes the reduction of oxo-phytodienoic acid to 10,11-dihydro-12-oxophytodienoic acid in jasmonic acid biosynthesis pathway [71]. Three of the ten *OPR* genes (*Os06g0216300*, *OsOPR1*; *Os06g0216200*, *OsOPR2*; *and Os06g0215600*, *OsOPR5*) in rice genome [72] were identified in this study. Among them, *OsOPR1* was reported to be transiently regulated by diverse environmental cues such as wounding, osmotic stress, UV-C irradiation and fungal elicitor [73]. Jasmonate ZIM domain proteins (JAZ) are key regulators of jasmonate hormonal responses by inhibiting DNA-binding transcription factors in the absence of jasmonic acid [74, 75]. ZIM (Zinc-finger protein expressed in inflorescence meristem) domain, also known as TIFY domain, is a plant specific transcription factor containing a core motif TIF[F/Y]XG [76, 77]. The identified two genes here were designated as *OsJAZ5* (*Os04g0395800*) and *OsJAZ8* (*Os09g0439200*) [78]. *OsJAZ8* was reported to participate in jasmonate-induced resistance to bacterial blight in rice [79]. Besides, we identified a gene encoding a jacalin-like lectin domain containing protein, *OsJAC1*, which was reported to be induced by methyl jasmonate and plays important roles in rice defense-related phenomena [80].

Finally we found a series of stress-related genes in the high C:low N list. *OsACA3* (*autoinhibited calcium ATPase 3*, *Os03g0203700*) encodes one of the fifteen plasma membrane Ca^2+^-ATPases in rice and is up-regulated under salt and cold stress [81]. Plasma membrane Ca^2+^-ATPases are transport proteins in the plasma membrane of cells and help in removal of Ca^2+^ from the cell [82]. In most cases, these proteins function in abiotic and biotic stress adaptation by regulating Ca^2+^ level within cells [83]. *OsPME* (*Os01g0788400*) encodes one of the 35 pectin methylesterases in rice [84]. Pectin methylesterases are ubiquitous enzymes that modify the degree of methylesterification of pectins, which are major components of cell walls in bacteria, fungi and plants [85]. In plants, *PME* genes belong to large multi-gene families and are primarily involved in developmental processes and plant-pathogen interactions [86, 87]. *OsAOX1B* (*Os04g0600300*) encodes one of the three alternative oxidases (AOXs) in rice genome [88]. AOX, located in the mitochondrial inner member, is a unique component in plant mitochondrial electron transport chain and catalyses the alternative respiratory pathway [89]. Most biotic and abiotic stresses, including pathogen or virus invasion, drought, high salinity, chilling, high light, high temperature, metal toxicity and nutrient limitation can increase the amounts of AOX, implying its involvement in stress tolerance [90, 91]. Furthermore, the protein content and capacity of AOX are up-regulated at low N condition in spinach (*Spinacia oleracea* L.) [92]. *OsPDR9* (*Os01g0609300*) encodes one of the 23 pleiotropic drug resistance (PDR)-like ATP-binding cassette (ABC) transporters in rice [93]. Plant PDR-like ABC transporters are expressed in response to various biotic and abiotic stresses and transport a diverse array of molecules across membranes [94]. *OsPDR9* was reported to be induced by jasmonic acid, heavy metals, high salt, hypoxic stress and redox perturbations, suggesting its role in stress responses [95] (Table 1).

Compared to the high number of high C:low N up-regulated genes, only 17 genes were found to be down-regulated by high C:low N and 10 up-regulated genes by low C:high N. All of the 17 high C:low N down-regulated genes have not been well studied and their functional information is hardly available. Among the low C:high N up-regulated genes, a GRAS transcription factor encoding gene (*Os05g0500600*) was identified. GRAS transcription factors constitute a large family of plant-specific proteins with at least 32 and 57 members in *Arabidopsis* and rice, respectively [96], and play critical roles in diverse biological processes, such as root development, gibberellic acid signaling and nodule symbiosis [97–99]. In addition, a chlorophyll a-b binding protein 2 (CAB2) encoding gene (*Os01g0720500*) was up-regulated by low C:high N. CAB, an antenna protein in the light harvesting complex, is essential for the uptake of light into photosystem II [100]. There are 17 genomic loci encoding for chlorophyll a-b binding proteins in rice [101]. Sugar has been reported to repress *CAB* gene expression in maize protoplasts [9]. In addition, *CAB* is not only down-regulated by sugar but also up-regulated by nitrogen and is considered as a marker gene of low C:high N status in *Arabidopsis* [26].

### Confirmation of microarray results by qRT-PCR analysis

**Fig 4:**
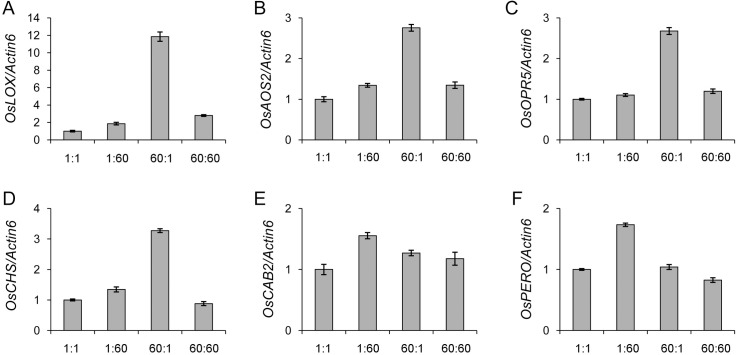
Validation of microarray results by qRT-PCR. (A) *OsLOX*; (B) *OsAOS2*; (C) *OsOPR5*; (D) *OsCHS*; (E) *OsCAB2*; (F) *OsPERO*. *Actin6* was used as the internal reference. The gray bars indicated the fold change of the genes between treatments (1:60, 60:1 and 60:60) and the control (1:1). Values are shown as means ± SDs from three technical replicates. A representative experiment of two biological replicates is shown.

To confirm the reliability of microarray results, we investigated the expression of some genes in rice seedlings with the same C:N treatments by qRT-PCR analysis. Three jasmonate synthetic genes (*OsLOX*, *OsAOS2* and *OsOPR5*), one chalcone synthase encoding gene (*OsCHS*), one chlorophyll a-b binding protein encoding gene (*OsCAB2*) and one peroxidase encoding gene (*OsPERO*) were selected. The three jasmonate synthetic genes as well as *OsCHS* were induced specifically by 60:1 treatment (Fig 4A, 4B, 4C and 4D) while *OsCAB2* and *OsPERO* were induced specifically by 1:60 treatment (Fig 4E and 4F). These expression patterns were consistent to the above analysis, demonstrating that our microarray results are reliable for the investigation of the genome-wide responsive genes to C:N treatments.

### Gene ontology (GO)-based functional categorization of imbalanced C:N responsive genes

**Fig 5:**
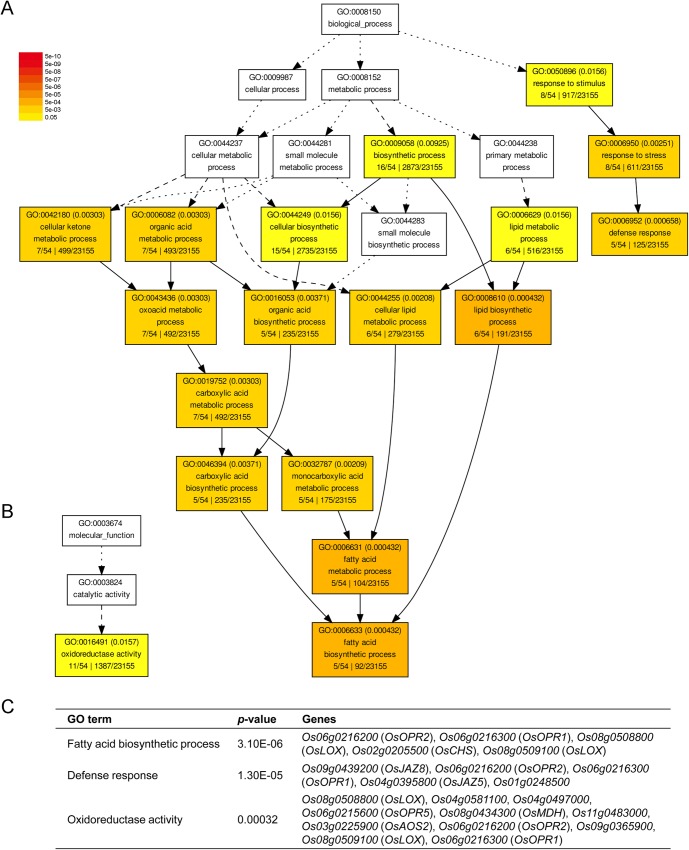
Gene ontology (GO) enrichment analysis of genes up-regulated by high C:low N. The differentially expressed probe sets were analyzed by SEA (singular enrichment analysis) using AgriGO, and the comparison is displayed in graphical mode. Each box contains GO term number, the false discovery rate (FDR) value, GO term and item number associated with the GO term in the query list and background as well as total number of query list and background. The degree of color saturation of a box is positively correlated to the enrichment level of the term (the yellow-to-red represents the term is up-regulated while non-significant terms are shown as white boxes). Solid, dashed and dotted lines represent two, one and zero enriched terms at both ends connected by the line, respectively. (A) Biological process category analysis of high C:low N up-regulated genes; (B) Molecular function category analysis of high C:low N up-regulated genes; (C) List of screened genes in “fatty acid biosynthesis”, “defense response” and “oxidoreductase activity” categories with *p*-values.

We employed GO category enrichment analysis to classify the biological functions of genes responsive to imbalanced C:N conditions. We searched the GO terms using AgriGO [32]. Using *p* ≤ 0.05 as cut-off, there were no significant GO categories for the genes down-regulated by high C:low N and neither those genes up-regulated by low C:high N. However, significantly enriched GO terms were identified for the genes up-regulated by high C:low N, which was listed in S3 File. Among the high C:low N up-regulated genes, several GO terms including oxoacid metabolism, organic acid biosynthesis, cellular lipid metabolism and lipid biosynthesis were significantly enriched (Fig 5A). First of all, the intersection of these GO terms was “fatty acid biosynthesis (GO: 0006952)”, which included four jasmonate synthesis genes and one chalcone synthesis gene mentioned above (Fig 5C). Second, eight high C:low N genes were enriched in GO term “response to stress”, among which two jasmonate synthetic genes and two ZIM domain containing genes as well as one pathogen-related protein encoding gene (*Os01g0248500*) were categorized in “defense response” process (GO: 0006633) (Fig 5A and 5C). The third enriched GO category was “oxidoreductase activity” (GO: 0016491) (Fig 5B). Oxidoreductase activity catalyzes an oxidation-reduction (redox) reaction, in which one substrate acts as electron donor and becomes oxidized while the other acts as electron acceptor and becomes reduced. Six jasmonate synthetic genes (two *OsLOX* genes, one *OsAOS* gene, and three *OsOPR* genes) and a malate dehydrogenase encoding gene were identified among the eleven genes in “oxidoreductase activity” category (Fig 5C).

### Coexpression analysis of high C:low N up-regulated genes

**Fig 6:**
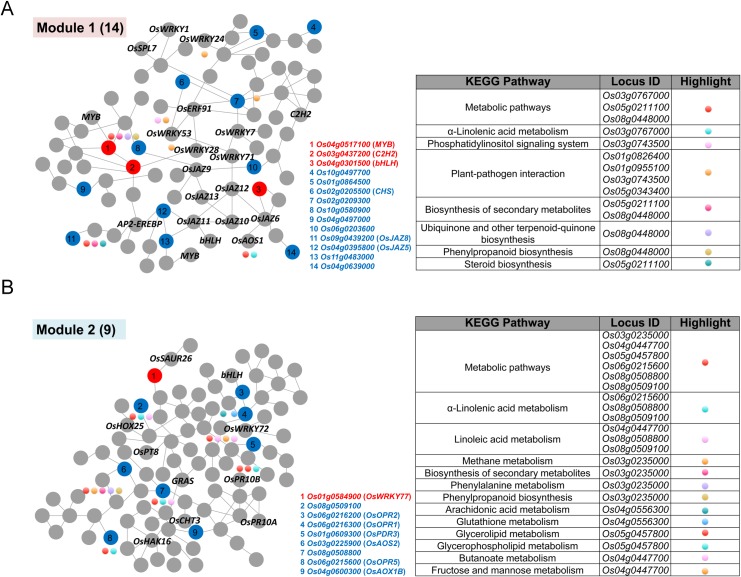
Coexpression network analysis of high C:low N up-regulated genes. (A) Module 1 extracted from coexpression analysis using 14 microarray identified genes. (B) Module 2 extracted from coexpression analysis using 9 microarray identified genes. Red and blue nodes indicate high C:low N up-regulated genes and the red ones are transcription factors. Other genes with known names or encode for transcription factors are marked on the nodes. Genes involved into KEGG pathways are marked with color dots beneath the nodes and the detailed information are listed on the tables.

Coexpression network analysis may provide clues to the functions of genes involved in specific biological processes. To gain better insights into the high C:low N up-regulated genes, coexpression network was developed using RiceFREND online program [33]. Two coexpression networks were formed with 111 and 116 edges in module 1 and module 2, respectively (Fig 6). In module 1, fourteen high C:low N up-regulated genes were the hubs connecting with other 72 genes (S4 File). Among these 86 genes, several ones encode for transcription factors, such as three *MYB* genes (*Os05g0553400*, *Os02g0624300* and *Os04g0517300*), six *WRKY* genes (*Os01g0246700*, *Os01g0826400*, *Os02g0181300*, *Os05g0343400*, *Os05g0537100* and *Os06g0649000*), two *C2H2* genes (*Os03g0437200* and *Os03g0820400*), two *bHLH* genes (*Os04g0301500* and *Os03g0741100*), two *AP2/EREBP* genes (*Os02g0654700*, *OsERF91*; *Os03g0860100*) and one *HSF* gene (*Heat stress transcription factor SPL7*, *Os05g0530400*). *OsSPL7* is a rice spotted leaf (lesion-mimic) gene encodes for a heat stress transcription factor [102]. Furthermore, AP2/EREBPs (APETALA2/ethylene-responsive element-binding proteins) belong to large family of transcription factors in plants and play important roles in the cross-talks among different stress signaling pathways [103]. The coexpression of these stress-related transcription factors are consistent with the above mentioned GO analysis category “response to stress” in high C:low N up-regulated genes. In addition, *OsAOS1* (*Os03g0767000*) and, surprisingly, eight of the fifteen *OsJAZ* genes in rice genome (*Os04g0395800*, *OsJAZ5*; *Os03g0402800*, *OsJAZ6*; *Os09g0439200*, *OsJAZ8*; *Os03g0180800*, *OsJAZ9*; *Os03g0181100*, *OsJAZ10*; *Os03g0180900*, *OsJAZ11*; *Os10g0392400*, *OsJAZ12*; *Os10g0391400*, *OsJAZ13*) were found in module 1 (Fig 6A, S4 File). Similar to *OsAOS2* discussed above, *OsAOS1* encodes for an allene oxide synthase, which serves as a key enzyme for the biosynthesis of jasmonic acid. The transcripts of *OsAOS1* were reported to be up-regulated transiently in response to light and wounding [104]. Among the *OsJAZ* genes in the network, *OsJAZ9* was reported to be a transcriptional regulator fine tuning the expression of JA-responsive genes involved in salt stress tolerance in rice [105]. In order to gain an overview of gene pathway networks, KEGG (Kyoto Encyclopedia of Genes and Genomics) analysis was performed using the online KEGG automatic annotation server. Eight categories were involved in the module 1. Plant-pathogen interaction and metabolic pathways (lipid metabolism and amino acid metabolism) were the most noteworthy ones with four and three genes, respectively (Fig 6A). These results are consistent with the GO categories “defense response” and “fatty acid biosynthetic process”, suggesting that high C:low N might serve as an inducer for defense responses, which involve certain fatty acid components.

In module 2, nine high C:low N up-regulated genes were the hubs connecting with other 89 genes (S4 File). Two *WRKY* genes (*Os01g0584900* and *Os11g0490900*), one *HD-Zip* gene (*Homeodomain-leucine zipper*, *Os09g0379600*), one *GRAS* gene (*Os11g0705200*) and one *bHLH* gene (*Os05g0455400*) were identified (Fig 6B, S4 File). HD-Zip transcription factors, which are unique in the plant kingdom, have diverse functions in plant development and often been implicated in stress adaptation [106]. The identified HD-Zip transcription factor is encoded by *OsHOX25* (*Homebox gene 25*). In rice, two of the 33 *OsHOX* genes have been studied in detail, among which *OsHOX4* was reported to be involved in drought stress [107] and *OsHOX22* functions in drought and salt stress [108]. In addition, the auxin responsive gene *OsSAUR26* (*Small auxin-up RNA 26*, *Os06g0671600*), the potassium (K^+^) transporter gene *OsHAK16* (*High-affinity K*^*+*^ *transporter 16*, *Os03g0575200*), the chitinase encoding gene *OsCHT3* (*Chitinase 3*, *Os06g0726100*) and two pathogenesis-related protein (PR protein) encoding genes *OsPR10A* (*Pathogenesis-related gene 10A*, *Os12g0555500*) and *OsPR10B* (*Pathogenesis-related gene 10B*, *Os12g0555200*) were identified in the module 2 coexpression network. Chitinase, a hydrolytic enzyme belonging to the PR proteins, could hydrolyze the cell wall chitin in most fungi and thus in plants chitinase acts in defense against fungal infection [109]. *OsCHT3*, also named as *PR3/OsChia1c*, functions as a defense-related gene in rice [110]. PR10 are small proteins with a molecular weight of 16 kDa. Many biotic or abiotic stresses have been shown to induce the expression of PR10 protein transcriptionally, suggesting their importance in plant defense responses. Three highly homologous *PR10* genes, *OsPR10A*, *OsPR10B* and *OsPR10C*, are known in rice, but *OsPR10C* appears to be a non-functional pseudogene [111]. These genes together with those in module 1 KEGG analysis (pathogen interaction) suggest enhanced defense responses under high C:low N conditions.

Network genes in module 2 fell into 13 categories of KEGG pathways including α-linolenic acid, linoleic acid metabolism and methane metabolism, etc (Fig 6B). It is known that jasmonate is derived from the unsaturated fatty acid α-linolenic acid (18:3), an octadecanoid abundant in the cellular membranes of higher plants [112]. Thus we propose that genes involved in α-linolenic acid metabolism in module 1 and module 2 networks might serve as jasmonate precursor synthetic genes. Taken together, the above coexpression network analysis of high C:low N up-regulated genes demonstrated that these genes function in jasmonate-mediated defense responses, suggesting that high C:low N imbalanced C:N nutrient status might serve as a special stress condition in plants.

## Discussion

Although it is controversial whether plants are able to sense and respond to C:N ratios [6, 7, 26, 113], it is well known that C and N nutrients interact with each other and that a balanced C:N availability is important for optimal plant growth and development. We speculated that plants would be able to sense and respond to imbalanced C:N availability by regulating genes involved in the C and N nutrient uptake and metabolism as well as genes dealing with the stresses caused by the imbalance of C:N. Our present findings based on global gene expression analysis provide strong evidence for this hypothesis [18, 21, 114]. Our analysis shows that both imbalanced high C:low N and low C:high N conditions induce changes in the expression of genes irrespective of absolute levels of C and N availabilities. In agreement with our hypothesis, these imbalanced C:N responsive genes include those involved C and N metabolism and stress responses.

In this study, we identified 63 up-regulated genes and 17 down-regulated genes by high C:low N. Low C:high N, however, was found to up-regulate the expression of 10 genes (Fig 3E). The larger number of high C:low N responsive genes compared with low C:high N responsive genes might come from the larger number of C regulated genes compared with N regulated genes (Fig 3A, 3B, 3C and 3D). As expected, some of these imbalanced C:N responsive genes are involved in the regulation of C and N assimilation/metabolism in the attempt for rice seedlings to reach C and N homeostasis (balance). Among the 10 up-regulated genes by low C:high N, *OsCAB2* is the homolog of *Arabidopsis CAB*, which has been demonstrated to be a low C:high N marker gene [26]. Several C:N signaling and metabolism related genes are also found to be regulated by high C:low N. Among them, two *OsCHS* genes are the homologs of *Arabidopsis CHS*, which is reported as high C:low N marker gene [26]. Increase in chalcone synthase levels is associated with higher anthocyanin accumulation and reduced chlorophyll contents and photosynthesis. *OsMDH* is another gene identified in high C:low N condition. Decreased MDH activity leads to enhanced photosynthetic performance in transgenic tomato plants [62]. If this is the case in rice, increased MDH levels would decrease the photosynthesis and contribute to the recovery of balanced C:N status under high C:low N condition. Furthermore, *OsLHT1* identified here might also serve as a regulator to maintain balanced C:N status, because overexpression of *LHT* resulted in increased amino acid uptake capacity and thus N use efficiency under limited inorganic N supply in *Arabidopsis* [55]. Last but not the least, one of the three *AOXs* in rice, *OsAOX1B*, is induced under the high C:low N condition. It was reported that under low N stress, the protein content and capacity of AOX would be preferentially increased and would efficiently consume excess carbohydrates, suppressing the rise of C:N ratio to maintain at a moderate level [92, 115]. A recent study also suggests that plants under disrupted C:N reduce the electron transport rate at PSII level and thus the excess of energy must be dissipated through alternative respiratory pathway [116]. In fact, the function of alternative respiratory pathway has long been controversial, for its electron transport from ubiquinone to water does not contribute to a transmembrane potential and wastes energies only [89]. Further studies found that AOX could be taken as a survival protein involved in the adaptation of stress conditions in plants [90, 117]. Despite AOX has been suggested to affect nitrate and carbon assimilation simultaneously [118], it is hard to distinguish the role of *OsAOX1B* identified here: dedicates into the recovery of balanced C:N or serves as a marker gene for stress condition?

We found that some jasmonate signaling components (two *OsLOX* genes, one *OsAOS* gene, three *OsOPR* genes and two *OsJAZ* genes), as well as several stress responsive genes, such as *OsMYB4*, *OsACA3* and *OsPDR9*, were up-regulated by high C:low N. GO category enrichment analysis found that these genes were mainly distributed in “fatty acid biosynthesis” and “defense response” processes. In addition, coexpression network analysis primarily identified two groups of new genes: jasmonate signaling components (e. g. *OsAOS1*, *OsJAZ* genes) and defense related genes (e. g. *OsSPL7*, *OsCHT3*, *OsPR10A* and *OsPR10B*). These results indicated that high C:low N status might result in defense responses regulated by jasmonate signaling in rice seedlings. C and N metabolism have been previously suggested to be involved in the plant defense responses [119, 120]. However, the mechanisms by which C:N contribute to plant immune responses are poorly understood. In spite of this, some preliminary studies are consistent with our observations. For example, sucrose treatment transcriptionally induces pathogenesis related genes [121] while N supply generally decreases defense responses, specifically, the high availability of N sources significantly increase the susceptibility of potato (*Solanum tuberosum*) to the oomycete *Phytophthora infestans* [122]. Moreover, defense related transcription factor genes *MYB51* and *WRKY33* are dramatically induced under high C:low N treatment in *Arabidopsis*, and a regulator of the C:N response, *Arabidopsis* ATL31 ubiquitin ligase, positively regulates the defense response against bacterial pathogens [123].

These findings raise an interesting question: What is the biological significance for the activation of defense responses by high C:low N treatment? Here we propose two possible explanations. First, increased jasmonate content or defense responses under high C:low N condition might be beneficial for the recovery of C:N balance. Given that C and N metabolites are apparently altered by pathogen infection [124] and long-distance transport of resources is one of the strategies in plant defense responses [125–127], the stress/defense responses under high C:low N condition might be related to the reallocation of CN nutrients. On the one hand, N transport and partitioning could be altered by jasmonate treatment [128, 129]. On the other hand, jasmonate mediated defense responses can decrease local photosynthetic rates by reducing photosynthetic electron transport and gas exchange [130–132] as well as accelerate the import of C from source leaves to sink tissues [126, 133]. The second explanation is that the induction of the defense responses might complement the defense ability, which is attenuated by the high growth rate under high C:low N condition. TOR proteins could sense nutrient conditions and control cell growth by regulating relevant transcriptional and translational processes. High C condition would activate TOR signaling pathway and thus the fast growth phenotype [5, 134]. According to the growth-defense hypothesis, fast-growing plant species should suffer more from pathogens than slower-growing species [135, 136]. In this case, the inductive defense responses under high C:low N condition might serve as a protective measure when most resources are invested into growth processes. Nonetheless, further studies are needed to investigate the potential relationship between C:N balancing mechanisms and jasmonate related stress/defense responses.

In addition to genes that can be categorized into functional pathways described above, imbalanced C:N conditions also induced a series of pioneer genes with unknown function and not associated with any genes with known GO categories. Almost all of the genes induced under low C:high N conditions (8 out 10) and 17 genes repressed under high C:low N conditions are in this category. We performed GO analysis to find some clues for the function of these genes, however, no enriched terms were found. Thus, these findings suggest possible novel mechanisms underlying the regulation of imbalanced C:N responses. Further studies should reveal these novel mechanisms and whether they are unique to plants accumulating high levels of carbohydrates such as cereal species.

## Supporting Information

S1 Fig Hierarchical cluster analysis of rice genes (probe sets) between CN treatments (1:1 and 60:1, 1:1 and 1:60, 1:60 and 60:60, 60:1 and 60:60). The log_2_ ratio values of probe sets were used for the analysis with R software. The colored bars represent the value (log_2_(fold change)) of the transcripts in each bin after CN treatments. Green represents down-regulated probe sets, red represents up-regulated probe sets, and dark indicates no significant difference in gene expression. (A) Hierarchical clustering of rice genes (probe sets) between 1:1 and 60:1; (B) Hierarchical clustering of rice genes (probe sets) between 1:1 and 1:60; (C) Hierarchical clustering of rice genes (probe sets) between 1:60 and 60:60; (D) Hierarchical clustering of rice genes (probe sets) between 60:1 and 60:60.
(TIF)

S1 File The raw microarray data for the rice expression profiling under four CN treatments. (XLS)

S2 File Differentially expressed gene lists between CN treatments. (XLS)

S3 File The GO category enrichment analysis results of up-regulated probe sets in high C:low N treatment. (XLSX)

S4 File Coexpression analysis results of module 1 and module 2 gene lists. (XLSX)

S1 Table List of gene specific primer sequences used in qRT-PCR. (DOCX)

## References

[1] Forchhammer K. Global carbon/nitrogen control by PII signal transduction in cyanobacteria: from signals to targets. FEMS Microbiol Rev. 2004;28(3):319–33. 10.1016/j.femsre.2003.11.001 15449606

[2] Jiang P, Ninfa AJ. *Escherichia coli* PII signal transduction protein controlling nitrogen assimilation acts as a sensor of adenylate energy charge *in vitro*. Biochem. 2007;46(45):12979–96. 10.1021/bi701062t 17939683

[3] Ninfa AJ, Atkinson MR. PII signal transduction proteins. Trends Microbiol. 2000;8(4):172–9. 10.1016/S0966-842X(00)01709-1. 10754576

[4] Rohde J, Heitman J, Cardenas ME. The TOR kinases link nutrient sensing to cell growth. J Biol Chem. 2001;276(13):9583–6. 10.1074/jbc.R000034200 . 11266435

[5] Dobrenel T, Caldana C, Hanson J, Robaglia C, Vincentz M, Veit B, et al TOR signaling and nutrient sensing. Annu Rev Plant Biol. 2016;67:261–85. 10.1146/annurev-arplant-043014-114648 . 26905651

[6] Gutierrez RA, Lejay LV, Dean A, Chiaromonte F, Shasha DE, Coruzzi GM. Qualitative network models and genome-wide expression data define carbon/nitrogen-responsive molecular machines in *Arabidopsis*. Genome Biol. 2007;8(1):R7 10.1186/gb-2007-8-1-r7 17217541 PMC1839130

[7] Palenchar PM, Kouranov A, Lejay LV, Coruzzi GM. Genome-wide patterns of carbon and nitrogen regulation of gene expression validate the combined carbon and nitrogen (CN)-signaling hypothesis in plants. Genome Biol. 2004;5(11):R91 10.1186/gb-2004-5-11-r91 15535867 PMC545782

[8] Stitt M, Krapp A. The interaction between elevated carbon dioxide and nitrogen nutrition: the physiological and molecular background. Plant Cell Environ. 1999;22(6):583–621. 10.1046/j.1365-3040.1999.00386.x

[9] Sheen J. Metabolic repression of transcription in higher plants. Plant Cell. 1990;2:1027–38. 10.1105/tpc.2.10.1027 2136626 PMC159951

[10] Ono K, Watanabe A. Levels of endogenous sugars, transcripts of *rbcS* and *rbcL*, and of RuBisco protein in senescing sunflower leaves. Plant Cell physiol. 1997;38(9):1032–8.

[11] Radin JW, Eidenbock MP. Carbon accumulation during photosynthesis in leaves of nitrogen- and phosphorus-stressed cotton. Plant Physiol. 1986;82:869–71. 16665124 10.1104/pp.82.3.869 PMC1056222

[12] Makino A, Osmond B. Effects of nitrogen nutrition on nitrogen partitioning between chloroplasts and mitochondria in pea and wheat. Plant Physiol. 1991;96:355–62. 16668193 10.1104/pp.96.2.355 PMC1080777

[13] Nunes-Nesi A, Fernie AR, Stitt M. Metabolic and signaling aspects underpinning the regulation of plant carbon nitrogen interactions. Mol Plant. 2010;3(6):973–96. 10.1093/mp/ssq049 . 20926550

[14] Krouk G, Ruffel S, Gutierrez RA, Gojon A, Crawford NM, Coruzzi GM, et al A framework integrating plant growth with hormones and nutrients. Trends Plant Sci. 2011;16(4):178–82. 10.1016/j.tplants.2011.02.004 . 21393048

[15] Bouguyon E, Gojon A, Nacry P. Nitrate sensing and signaling in plants. Semin Cell Dev Biol. 2012;23(6):648–54. 10.1016/j.semcdb.2012.01.004 . 22273693

[16] Zhang H, Jennings A, Barlow PW, Forde BG. Dual pathways for regulation of root branching by nitrate. Proc Natl Acad Sci U S A. 1999;96(11):6529–34. . 10339622 10.1073/pnas.96.11.6529 PMC26916

[17] Yates RC, Curtis JT. The effect of sucrose and other factors on the shoot-root ratio of orchid seedlings. Am J Bot. 1949;36(5):390–6. 10.2307/2437736 18120725

[18] Sun WF, Huang AB, Sang YY, Fu Y, Yang ZB. Carbon–nitrogen interaction modulates plant growth and expression of metabolic genes in rice. J Plant Growth Regul. 2013;32(3):575–84. 10.1007/s00344-013-9324-x

[19] Chen X, Yao Q, Gao X, Jiang C, Harberd NP, Fu X. Shoot-to-root mobile transcription factor HY5 coordinates plant carbon and nitrogen acquisition. Curr Biol. 2016;26(5):640–6. 10.1016/j.cub.2015.12.066 . 26877080

[20] Foyer CH, Noctor G, Verrier P. Photosynthetic carbon–nitrogen interactions: modelling inter-pathway control and signalling Annual Plant Reviews Volume 22: Control of Primary Metabolism in Plants: Blackwell Publishing Ltd; 2007 p. 325–47.

[21] Zheng ZL. Carbon and nitrogen nutrient balance signaling in plants. Plant Signal Behav. 2009;4(7):584–91. 10.4161/psb.4.7.8540 19820356 PMC2710548

[22] Vincentz M, Moureaux T, Leydecker MT, Vaucheret H, Caboche M. Regulation of nitrate and nitrite reductase expression in *Nicotiana plumbaginifolia* leaves by nitrogen and carbon metabolites. Plant J. 1993;3(2):315–24. 8220446 10.1111/j.1365-313x.1993.tb00183.x

[23] Klein D, Morcuende R, Stitt M, Krapp A. Regulation of nitrate reductase expression in leaves by nitrate and nitrogen metabolism is completely overridden when sugars fall below a critical level. Plant Cell Environ. 2000;23(8):863–71. 10.1046/j.1365-3040.2000.00593.x

[24] Tsukaya H, Ohshima T, Naito S, Chino M, Komeda Y. Sugar-dependent expression of the *CHS*-a gene for chalcone synthase from petunia in transgenic *Arabidopsis*. Plant Physiol. 1991;97:1414–21. 16668565 10.1104/pp.97.4.1414 PMC1081180

[25] Coronado C, Zuanazzi JAS, Sallaud C, Quirion JC, Esnault R, Husson HP, et al Alfalfa root flavonoid production is nitrogen regulated. Plant Physiol. 1995;108:533–42. 12228491 10.1104/pp.108.2.533 PMC157372

[26] Martin T, Oswald O, Graham IA. *Arabidopsis* seedling growth, storage lipid mobilization, and photosynthetic gene expression are regulated by carbon:nitrogen availability. Plant Physiol. 2002;128(2):472–81. 10.1104/pp.010475 11842151 PMC148910

[27] Kang J, Turano FJ. The putative glutamate receptor 1.1 (AtGLR1.1) functions as a regulator of carbon and nitrogen metabolism in *Arabidopsis thaliana*. Proc Natl Acad Sci U S A. 2003;100(11):6872–7. 10.1073/pnas.1030961100 12738881 PMC164539

[28] Gao P, Xin Z, Zheng ZL. The *OSU1/QUA2/TSD2*-encoded putative methyltransferase is a critical modulator of carbon and nitrogen nutrient balance response in *Arabidopsis*. PloS One. 2008;3(1):e1387 10.1371/journal.pone.0001387 18167546 PMC2148111

[29] Sato T, Maekawa S, Yasuda S, Sonoda Y, Katoh E, Ichikawa T, et al CNI1/ATL31, a RING-type ubiquitin ligase that functions in the carbon/nitrogen response for growth phase transition in *Arabidopsis* seedlings. Plant J. 2009;60(5):852–64. 10.1111/j.1365-313X.2009.04006.x . 19702666

[30] Aoyama S, Huarancca Reyes T, Guglielminetti L, Lu Y, Morita Y, Sato T, et al Ubiquitin ligase ATL31 functions in leaf senescence in response to the balance between atmospheric CO2 and nitrogen availability in *Arabidopsis*. Plant Cell Physiol. 2014;55(2):293–305. 10.1093/pcp/pcu002 24399238 PMC3913444

[31] Gamborg OL, Miller RA, Ojima K. Nutrient requirements of suspension cultures of soybean root cells. Exp Cell Res. 1968;50(1):151–8. 10.1016/0014-4827(68)90403-5. 5650857

[32] Du Z, Zhou X, Ling Y, Zhang Z, Su Z. AgriGO: a GO analysis toolkit for the agricultural community. Nucleic Acids Res. 2010;38:W64–70. 10.1093/nar/gkq310 20435677 PMC2896167

[33] Sato Y, Namiki N, Takehisa H, Kamatsuki K, Minami H, Ikawa H, et al RiceFREND: a platform for retrieving coexpressed gene networks in rice. Nucleic Acids Res. 2013;41(Database issue):D1214–21. 10.1093/nar/gks1122 23180784 PMC3531108

[34] Yanagisawa S, Akiyama A, Kisaka H, Uchimiya H, Miwa T. Metabolic engineering with Dof1 transcription factor in plants: improved nitrogen assimilation and growth under low-nitrogen conditions. Proc Natl Acad Sci U S A. 2004;101(20):7833–8. 10.1073/pnas.0402267101 15136740 PMC419692

[35] Champigny ML. Integration of photosynthetic carbon and nitrogen metabolism in higher plants. Photosynth Res. 1995;46(1):117–27. 10.1007/bf00020422 24301574

[36] Eulgem T, Rushton PJ, Robatzek S, Somssich IE. The WRKY superfamily of plant transcription factors. Trends Plant Sci. 2000;5(5):199–205. 10785665 10.1016/s1360-1385(00)01600-9

[37] Pandey SP, Somssich IE. The role of WRKY transcription factors in plant immunity. Plant Physiol. 2009;150(4):1648–55. 10.1104/pp.109.138990 19420325 PMC2719123

[38] Ross CA, Liu Y, Shen QJ. The WRKY gene family in rice (*Oryza sativa*). J Integr Plant Biol. 2007;49(6):827–42. 10.1111/j.1672-9072.2007.00504.x

[39] Englbrecht CC, Schoof H, Bohm S. Conservation, diversification and expansion of C2H2 zinc finger proteins in the *Arabidopsis thaliana* genome. BMC Genomics. 2004;5(1):39 10.1186/1471-2164-5-39 15236668 PMC481060

[40] Ciftci-Yilmaz S, Mittler R. The zinc finger network of plants. Cell Mol Life Sci. 2008;65(7–8):1150–60. 10.1007/s00018-007-7473-4 . 18193167 PMC11131624

[41] Yang W, Yoon J, Choi H, Fan Y, Chen R, An G. Transcriptome analysis of nitrogen-starvation-responsive genes in rice. BMC Plant Biol. 2015;15:31 10.1186/s12870-015-0425-5 25644226 PMC4333837

[42] Carretero-Paulet L, Galstyan A, Roig-Villanova I, Martinez-Garcia JF, Bilbao-Castro JR, Robertson DL. Genome-wide classification and evolutionary analysis of the bHLH family of transcription factors in *Arabidopsis*, poplar, rice, moss, and algae. Plant Physiol. 2010;153(3):1398–412. 10.1104/pp.110.153593 20472752 PMC2899937

[43] Li X, Duan X, Jiang H, Sun Y, Tang Y, Yuan Z, et al Genome-wide analysis of basic/helix-loop-helix transcription factor family in rice and *Arabidopsis*. Plant Physiol. 2006;141(4):1167–84. 10.1104/pp.106.080580 16896230 PMC1533929

[44] Dubos C, Stracke R, Grotewold E, Weisshaar B, Martin C, Lepiniec L. MYB transcription factors in *Arabidopsis*. Trends Plant Sci. 2010;15(10):573–81. 10.1016/j.tplants.2010.06.005 . 20674465

[45] Paz-Ares J, Ghosal D, Wienand U, Peterson P, Saedler H. The regulatory *c1* locus of *Zea mays* encodes a protein with homology to *myb* proto-oncogene products and with structural similarities to transcriptional activators. EMBO J. 1987;6(12):3553–8. 3428265 10.1002/j.1460-2075.1987.tb02684.x PMC553820

[46] Katiyar A, Smita S, Lenka SK, Rajwanshi R, Chinnusamy V, Bansal KC. Genome-wide classification and expression analysis of MYB transcription factor families in rice and *Arabidopsis*. BMC Genomics. 2012;13(544). 10.1186/1471-2164-13-544 PMC3542171 23050870

[47] Soltesz A, Vagujfalvi A, Rizza F, Kerepesi I, Galiba G, Cattivelli L, et al The rice *Osmyb4* gene enhances tolerance to frost and improves germination under unfavourable conditions in transgenic barley plants. J Appl Genet. 2012;53(2):133–43. 10.1007/s13353-011-0081-x . 22246661

[48] Vannini C, Locatelli F, Bracale M, Magnani E, Marsoni M, Osnato M, et al Overexpression of the rice *Osmyb4* gene increases chilling and freezing tolerance of *Arabidopsis thaliana* plants. Plant J. 2004;37:115–27. 14675437 10.1046/j.1365-313x.2003.01938.x

[49] Vannini C, Campa M, Iriti M, Genga A, Faoro F, Carravieri S, et al Evaluation of transgenic tomato plants ectopically expressing the rice *Osmyb4* gene. Plant Sci. 2007;173(2):231–9. 10.1016/j.plantsci.2007.05.007

[50] Pasquali G, Biricolti S, Locatelli F, Baldoni E, Mattana M. *Osmyb4* expression improves adaptive responses to drought and cold stress in transgenic apples. Plant Cell Rep. 2008;27(10):1677–86. 10.1007/s00299-008-0587-9 . 18679687

[51] Laura M, Consonni R, Locatelli F, Fumagalli E, Allavena A, Coraggio I, et al Metabolic response to cold and freezing of *Osteospermum ecklonis* overexpressing *Osmyb4*. Plant Physiol Biochem. 2010;48(9):764–71. 10.1016/j.plaphy.2010.06.003 . 20619667

[52] Chen LS, Bush DR. LHT1, a lysine- and histidine-specific amino acid transporter in *arabidopsis*. Plant Physiol. 1997;115:1127–34. 9390441 10.1104/pp.115.3.1127 PMC158577

[53] Lee YH, Tegeder M. Selective expression of a novel high-affinity transport system for acidic and neutral amino acids in the tapetum cells of *Arabidopsis* flowers. Plant J. 2004;40(1):60–74. 10.1111/j.1365-313X.2004.02186.x . 15361141

[54] Svennerstam H, Ganeteg U, Bellini C, Nasholm T. Comprehensive screening of *Arabidopsis* mutants suggests the lysine histidine transporter 1 to be involved in plant uptake of amino acids. Plant Physiol. 2007;143(4):1853–60. 10.1104/pp.106.092205 17293438 PMC1851813

[55] Hirner A, Ladwig F, Stransky H, Okumoto S, Keinath M, Harms A, et al *Arabidopsis* LHT1 is a high-affinity transporter for cellular amino acid uptake in both root epidermis and leaf mesophyll. Plant Cell. 2006;18(8):1931–46. 10.1105/tpc.106.041012 16816136 PMC1533986

[56] Tegeder M, Ward JM. Molecular evolution of plant AAP and LHT amino acid transporters. Front Plant Sci. 2012;3:21 10.3389/fpls.2012.00021 22645574 PMC3355764

[57] Zhao H, Ma H, Yu L, Wang X, Zhao J. Genome-wide survey and expression analysis of amino acid transporter gene family in rice (*Oryza sativa* L.). PLoS One. 2012;7(11):e49210 10.1371/journal.pone.0049210 23166615 PMC3499563

[58] Reimold U, Kroger M, Kreuzaler F, Hahlbrock K. Coding and 3' non-coding nucleotide sequence of chalcone synthase mRNA and assignment of amino acid sequence of the enzyme. EMBO J. 1983;2(10):1801–5. 16453477 10.1002/j.1460-2075.1983.tb01661.x PMC555362

[59] Zhou B, Wang Y, Zhan Y, Li Y, Kawabata S. Chalcone synthase family genes have redundant roles in anthocyanin biosynthesis and in response to blue/UV-A light in turnip (*Brassica rapa*; Brassicaceae). Am J Bot. 2013;100(12):2458–67. 10.3732/ajb.1300305 . 24197179

[60] Feyissa DN, Lovdal T, Olsen KM, Slimestad R, Lillo C. The endogenous *GL3*, but not *EGL3*, gene is necessary for anthocyanin accumulation as induced by nitrogen depletion in *Arabidopsis* rosette stage leaves. Planta. 2009;230(4):747–54. 10.1007/s00425-009-0978-3 . 19621239

[61] Goward CR, Nicholis DJ. Malate dehydrogenase: a model for structure, evolution, and catalysis. Protein Sci. 1994;3:1883–8. 10.1002/pro.5560031027 7849603 PMC2142602

[62] Nunes-Nesi A, Carrari F, Lytovchenko A, Smith AM, Loureiro ME, Ratcliffe RG, et al Enhanced photosynthetic performance and growth as a consequence of decreasing mitochondrial malate dehydrogenase activity in transgenic tomato plants. Plant Physiol. 2005;137(2):611–22. 10.1104/pp.104.055566 15665243 PMC1065362

[63] Gaffney BJ. Lipoxygenases structural principles and spectroscopy. Annu Rev Biophys Biomol Struct. 1996;25:431–59. 10.1146/annurev.bb.25.060196.002243 8800477

[64] Truner JG, Ellis c, Devoto A. The jasmonate signal pathway. Plant Cell. 2002:S153–S64. 10.1105/tpc.000679 12045275 PMC151253

[65] Porta H, Sosa MR. Plant lipoxygenases. Physiological and molecular features. Plant Physiol. 2002;130:15–21. 10.1104/pp.010787 12226483 PMC1540254

[66] Umate P. Genome-wide analysis of lipoxygenase gene family in *Arabidopsis* and rice. Plant Signal Behav. 2011;6(3):335–8. 10.4161/psb.6.3.13546 21336026 PMC3142411

[67] Grechkin A. Recent developments in biochemistry of the plant lipoxygenase pathway. Prog Lipid Res. 1998;37(5):317–52. 10209652 10.1016/s0163-7827(98)00014-9

[68] Agrawal GK, Tamogami S, Han O, Iwahashi H, Rakwal R. Rice octadecanoid pathway. Biochem Biophys Res Commun. 2004;317(1):1–15. 10.1016/j.bbrc.2004.03.020 . 15047141

[69] Agrawal GK, Rakwal R, Jwa NS, Han KS, Agrawal VP. Molecular cloning and mRNA expression analysis of the first rice jasmonate biosynthetic pathway gene allene oxide synthase. Plant Physiol Biochem. 2002;40:771–82.

[70] Yoeun S, Kim J-I, Han O. Cellular localization and detergent dependent oligomerization of rice allene oxide synthase-1. J Plant Res. 2014 10.1007/s10265-014-0670-y 25326901

[71] Vick BA, Zimmerman DC. Characterization of 12-oxo-phytodienoic acid reductase in corn. Plant Physiol. 1986;80:202–5. 16664582 10.1104/pp.80.1.202 PMC1075082

[72] Tani T, Sobajima H, Okada K, Chujo T, Arimura S, Tsutsumi N, et al Identification of the *OsOPR7* gene encoding 12-oxophytodienoate reductase involved in the biosynthesis of jasmonic acid in rice. Planta. 2008;227(3):517–26. 10.1007/s00425-007-0635-7 . 17938955

[73] Agrawal GK, Jwa N-S, Shibato J, Han O, Iwahashi H, Rakwal R. Diverse environmental cues transiently regulate *OsOPR1* of the “octadecanoid pathway” revealing its importance in rice defense/stress and development. Biochem Biophys Res Commun. 2003;310(4):1073–82. 10.1016/j.bbrc.2003.09.123 14559225

[74] Chini A, Fonseca S, Fernandez G, Adie B, Chico JM, Lorenzo O, et al The JAZ family of repressors is the missing link in jasmonate signalling. Nature. 2007;448(7154):666–71. 10.1038/nature06006 . 17637675

[75] Thines B, Katsir L, Melotto M, Niu Y, Mandaokar A, Liu G, et al JAZ repressor proteins are targets of the SCF(COI1) complex during jasmonate signalling. Nature. 2007;448(7154):661–5. 10.1038/nature05960 . 17637677

[76] Nishii A, Takemura M, Fujita H, Shikata M, Yokota A, Kohchi T. Characterization of a novel gene encoding a putative single zinc-finger protein, ZIM, expressed during the reproductive phase in *Arabidopsis thaliana*. Biosci Biotechnol Biochem. 2000;64(7):1402–9. 10.1271/bbb.64.1402 10945256

[77] Vanholme B, Grunewald W, Bateman A, Kohchi T, Gheysen G. The tify family previously known as ZIM. Trends Plant Sci. 2007;12(6):239–44. 10.1016/j.tplants.2007.04.004 . 17499004

[78] Ye H, Du H, Tang N, Li X, Xiong L. Identification and expression profiling analysis of TIFY family genes involved in stress and phytohormone responses in rice. Plant Mol Biol. 2009;71(3):291–305. 10.1007/s11103-009-9524-8 . 19618278

[79] Yamada S, Kano A, Tamaoki D, Miyamoto A, Shishido H, Miyoshi S, et al Involvement of OsJAZ8 in jasmonate-induced resistance to bacterial blight in rice. Plant Cell Physiol. 2012;53(12):2060–72. 10.1093/pcp/pcs145 . 23104764

[80] Jiang JF, Han Y, Xing LJ, Xu YY, Xu ZH, Chong K. Cloning and expression of a novel cDNA encoding a mannose-specific jacalin-related lectin from *Oryza sativa*. Toxicon. 2006;47(1):133–9. 10.1016/j.toxicon.2005.10.010 . 16359716

[81] Singh A, Kanwar P, Yadav AK, Mishra M, Jha SK, Baranwal V, et al Genome-wide expressional and functional analysis of calcium transport elements during abiotic stress and development in rice. FEBS J. 2014;281(3):894–915. 10.1111/febs.12656 . 24286292

[82] Briskin D. Ca^2+^-translocating ATPase of the plant plasma membrane. Plant Physiol. 1990;94:397–400. 16667727 10.1104/pp.94.2.397 PMC1077244

[83] Huda KM, Yadav S, Akhter Banu MS, Trivedi DK, Tuteja N. Genome-wide analysis of plant-type II Ca^2+^ ATPases gene family from rice and *Arabidopsis*: potential role in abiotic stresses. Plant Physiol Biochem. 2013;65:32–47. 10.1016/j.plaphy.2013.01.002 . 23416494

[84] Pelloux J, Rusterucci C, Mellerowicz EJ. New insights into pectin methylesterase structure and function. Trends Plant Sci. 2007;12(6):267–77. 10.1016/j.tplants.2007.04.001 . 17499007

[85] Micheli F. Pectin methylesterases: cell wall enzymes with important roles in plant physiology. Trends Plant Sci. 2001;6(9):414–9. 11544130 10.1016/s1360-1385(01)02045-3

[86] Tian GW, Chen MH, Zaltsman A, Citovsky V. Pollen-specific pectin methylesterase involved in pollen tube growth. Dev Biol. 2006;294(1):83–91. 10.1016/j.ydbio.2006.02.026 . 16564517

[87] Lionetti V, Cervone F, Bellincampi D. Methyl esterification of pectin plays a role during plant-pathogen interactions and affects plant resistance to diseases. J Plant Physiol. 2012;169(16):1623–30. 10.1016/j.jplph.2012.05.006 . 22717136

[88] Saika H, Ohtsu K, Hamanaka S, Nakazono M, Tsutsumi N, Hirai A. *AOX1c*, a novel rice gene for alternative oxidase; comparison with rice *AOX1a* and *AOX1b*. Genes Genet Syst. 2002;77:31–8. 12036102 10.1266/ggs.77.31

[89] Vanlerberghe GC, McIntosh L. Aleternative oxidase: from gene to function. Annu Rev Plant Physiol Plant Mol Biol. 1997;48:703–34. 10.1146/annurev.arplant.48.1.703 15012279

[90] Van Aken O, Giraud E, Clifton R, Whelan J. Alternative oxidase: a target and regulator of stress responses. Physiol Plant. 2009;137(4):354–61. 10.1111/j.1399-3054.2009.01240.x . 19470093

[91] Feng H, Guan D, Sun K, Wang Y, Zhang T, Wang R. Expression and signal regulation of the alternative oxidase genes under abiotic stresses. Acta Biochim Biophys Sin. 2013;45(12):985–94. 10.1093/abbs/gmt094 . 24004533

[92] Noguchi KO, Terashima I. Responses of spinach leaf mitochondria to low N availability. Plant Cell Environ. 2006;29(4):710–9. 10.1111/j.1365-3040.2005.01457.x 17080620

[93] Jasinski M, Ducos E, Martinoia E, Boutry M. The ATP-binding cassette transporters: structure, function, and gene family comparison between rice and *Arabidopsis*. Plant Physiol. 2003;131(3):1169–77. 10.1104/pp.102.014720 12644668 PMC1540298

[94] Nuruzzaman M, Zhang R, Cao H-Z, Luo Z-Y. Plant pleiotropic drug resistance transporters: transport mechanism, gene expression, and function. J Integr Plant Biol. 2014;56(8):729–40. 10.1111/jipb.12196 24645852

[95] Moons A. *Ospdr9*, which encodes a PDR-type ABC transporter, is induced by heavy metals, hypoxic stress and redox perturbations in rice roots1. FEBS Lett. 2003;553(3):370–6. 10.1016/s0014-5793(03)01060-3 14572653

[96] Tian C, Wan P, Sun SH, Li JY, Chen MS. Genome-wide analysis of the *GRAS* gene family in rice and *Arabidopsis*. Plant Mol Biol. 2004;54:519–32. 10.1023/B:PLAN.0000038256.89809.57 15316287

[97] Itoh H, Shimada A, Ueguchi-Tanaka M, Kamiya N, Hasegawa Y, Ashikari M, et al Overexpression of a GRAS protein lacking the DELLA domain confers altered gibberellin responses in rice. Plant J. 2005;44(4):669–79. 10.1111/j.1365-313X.2005.02562.x . 16262715

[98] Heckmann AB, Lombardo F, Miwa H, Perry JA, Bunnewell S, Parniske M, et al *Lotus japonicus* nodulation requires two GRAS domain regulators, one of which is functionally conserved in a non-legume. Plant Physiol. 2006;142(4):1739–50. 10.1104/pp.106.089508 17071642 PMC1676053

[99] Miyashima S, Hashimoto T, Nakajima K. ARGONAUTE1 acts in *Arabidopsis* root radial pattern formation independently of the SHR/SCR pathway. Plant Cell Physiol. 2009;50(3):626–34. 10.1093/pcp/pcp020 . 19188262

[100] Yang DH, P H., Andersson B. The N-terminal domain of the light-harvesting chlorophyll a/b-binding protein complex (LHCII) is essential for its acclimative proteolysis. FEBS J. 2000;466:385–8. 10.1016/s0014-5793(00)01107-8 10682866

[101] Umate P. Genome-wide analysis of the family of light-harvesting chlorophyll a/b-binding proteins in *Arabidopsis* and rice. Plant Signal Behav. 2010;5(12):1537–42. 10.4161/psb.5.12.13410 21512324 PMC3115097

[102] Yamanouchi U, Yano M, Lin H, Ashikari M, Yamada K. A rice spotted leaf gene, *Spl7*, encodes a heat stress transcription factor protein. Proc Natl Acad Sci U S A. 2002;99(11):7530–5. 10.1073/pnas.112209199 12032317 PMC124274

[103] Sharoni AM, Nuruzzaman M, Satoh K, Shimizu T, Kondoh H, Sasaya T, et al Gene structures, classification and expression models of the AP2/EREBP transcription factor family in rice. Plant Cell Physiol. 2011;52(2):344–60. 10.1093/pcp/pcq196 . 21169347

[104] Haga K, Iino M. Phytochrome-mediated transcriptional up-regulation of *ALLENE OXIDE SYNTHASE* in rice seedlings. Plant Cell Physiol. 2004;45(2):119–28. 14988482 10.1093/pcp/pch025

[105] Wu H, Ye H, Yao R, Zhang T, Xiong L. OsJAZ9 acts as a transcriptional regulator in jasmonate signaling and modulates salt stress tolerance in rice. Plant Sci. 2015;232:1–12. 10.1016/j.plantsci.2014.12.010 . 25617318

[106] Wang H, Lin J, Li XG, Chang Y. Genome-wide identification of pear HD-Zip gene family and expression patterns under stress induced by drought, salinity, and pathogen. Acta Physiol Plant. 2015;37(9). 10.1007/s11738-015-1933-5

[107] Agalou A, Purwantomo S, Overnas E, Johannesson H, Zhu X, Estiati A, et al A genome-wide survey of HD-Zip genes in rice and analysis of drought-responsive family members. Plant Mol Biol. 2008;66(1–2):87–103. 10.1007/s11103-007-9255-7 . 17999151

[108] Zhang S, Haider I, Kohlen W, Jiang L, Bouwmeester H, Meijer AH, et al Function of the HD-Zip I gene *Oshox22* in ABA-mediated drought and salt tolerances in rice. Plant Mol Biol. 2012;80(6):571–85. 10.1007/s11103-012-9967-1 . 23109182

[109] Collinge DB, Kragh KM, Mikkelsen JD, Nielsen KK, Rasmussen U, Vad K. Plant chitinases. Plant J. 1993;3(1):31–40. 10.1046/j.1365-313X.1993.t01-1-00999.x 8401605

[110] Yokotani N, Tsuchida-Mayama T, Ichikawa H, Mitsuda N, Ohme-Takagi M, Kaku H, et al OsNAC111, a blast disease-responsive transcription factor in rice, positively regulates the expression of defense-related genes. Mol Plant Microbe Interact. 2014;27(10):1027–34. 10.1094/MPMI-03-14-0065-R . 25014590

[111] Hashimoto M, Kisseleva L, Sawa S, Furukawa T, Komatsu S, Koshiba T. A novel rice PR10 protein, RSOsPR10, specifically induced in roots by biotic and abiotic stresses, possibly via the jasmonic acid signaling pathway. Plant Cell Physiol. 2004;45(5):550–9. 10.1093/pcp/pch063 15169937

[112] Gfeller A, Dubugnon L, Liechti R, Farmer EE. Jasmonate Biochemical Pathway. Sci Signal. 2010;3(109):cm3–cm. 10.1126/scisignal.3109cm3 20159849

[113] Coruzzi GM, Zhou L. Carbon and nitrogen sensing and signaling in plants emerging ‘matrix effects’. Curr Opin Plant Biol. 2001;4:247–53. 11312136 10.1016/s1369-5266(00)00168-0

[114] Chen D, Wang S, Xiong B, Cao B, Deng X. Carbon/nitrogen imbalance associated with drought-induced leaf senescence in *Sorghum bicolor*. PLoS One. 2015;10(8):e0137026 10.1371/journal.pone.0137026 26317421 PMC4552878

[115] Sieger SM, Kristensen BK, Robson CA, Amirsadeghi S, Eng EW, Abdel-Mesih A, et al The role of alternative oxidase in modulating carbon use efficiency and growth during macronutrient stress in tobacco cells. J Exp Bot. 2005;56(416):1499–515. 10.1093/jxb/eri146 . 15824074

[116] Huarancca Reyes T, Scartazza A, Lu Y, Yamaguchi J, Guglielminetti L. Effect of carbon/nitrogen ratio on carbohydrate metabolism and light energy dissipation mechanisms in *Arabidopsis thaliana*. Plant Physiol Biochem. 2016;105:195–202. 10.1016/j.plaphy.2016.04.030 . 27108206

[117] Juszczuk IM, Rychter AM. Alternative oxidase in higher plants. Acta Biochim Pol. 2003;50(4):1257–71. 14740012

[118] Gandin A, Denysyuk M, Cousins AB. Disruption of the mitochondrial alternative oxidase (AOX) and uncoupling protein (UCP) alters rates of foliar nitrate and carbon assimilation in *Arabidopsis thaliana*. J Exp Bot. 2014;65(12):3133–42. 10.1093/jxb/eru158 24799562 PMC4071831

[119] Herbers K, Takahata Y, Melzer M, Mock HP, Hajirezaei M, Sonnewald U. Regulation of carbohydrate partitioning during the interaction of potato virus Y with tobacco. Mol Plant Pathol 2000;1(1):51–9. 10.1046/j.1364-3703.2000.00007.x 20572950

[120] Mittelstraß K, Treutter D, Pleßl M, Heller W, Elstner EF, Heiser I. Modification of primary and secondary metabolism of potato plants by nitrogen application differentially affects resistance to *phytophthora infestans* and *alternaria solani*. Plant Biol. 2006;8(5):653–61. 10.1055/s-2006-924085 16821190

[121] Thibaud MC, Gineste S, Nussaume L, Robaglia C. Sucrose increases pathogenesis-related PR-2 gene expression in *Arabidopsis thaliana* through an SA-dependent but NPR1-independent signaling pathway. Plant Physiol Biochem. 2004;42(1):81–8. 10.1016/j.plaphy.2003.10.012 . 15061088

[122] Ros B, Mohler V, Wenzel G, Thümmler F. Phytophthora infestans-triggered response of growth- and defense-related genes in potato cultivars with different levels of resistance under the influence of nitrogen availability. Physiol Plant. 2008;133(2):386–96. 10.1111/j.1399-3054.2008.01048.x 18282193

[123] Maekawa S, Sato T, Asada Y, Yasuda S, Yoshida M, Chiba Y, et al The *Arabidopsis* ubiquitin ligases ATL31 and ATL6 control the defense response as well as the carbon/nitrogen response. Plant Mol Biol. 2012;79(3):217–27. 10.1007/s11103-012-9907-0 . 22481162

[124] Molitor A, Zajic D, Voll LM, Pons-Kühnemann J, Samans B, Kogel K-H, et al Barley leaf transcriptome and metabolite analysis reveals new aspects of compatibility and *piriformospora indica*–mediated systemic induced resistance to powdery mildew. Mol Plant Microbe Interact. 2011;24(12):1427–39. 10.1094/MPMI-06-11-0177 21830949

[125] Matyssek R, Schnyder H, Munch JC, Oßwald W, Pretzsch H, Treutter D. Resource allocation in plants—the balance between resource sequestration and retention. Plant Biol. 2005;7(6):557–9. 10.1055/s-2005-873000 16388460

[126] Appel HM, Arnold TM, Schultz JC. Effects of jasmonic acid, branching and girdling on carbon and nitrogen transport in poplar. New Phytol. 2012;195(2):419–26. 10.1111/j.1469-8137.2012.04171.x . 22621389

[127] Arnold TM, Appel HM, Schultz JC. Is polyphenol induction simply a result of altered carbon and nitrogen accumulation. Plant Signal Behav. 2012;7(11):1498–500. 10.4161/psb.21900 22960757 PMC3548879

[128] Meuriot F, Noquet C, Avice J-C, Volenec JJ, Cunningham SM, Sors TG, et al Methyl jasmonate alters N partitioning, N reserves accumulation and induces gene expression of a 32-kDa vegetative storage protein that possesses chitinase activity in *Medicago sativa* taproots. Physiol Plant. 2004;120(1):113–23. 10.1111/j.0031-9317.2004.0210.x 15032883

[129] Rossato L, MacDuff JH, Laine P, Le Deunff E, Ourry A. Nitrogen storage and remobilization in *Brassica napus* L. during the growth cycle: effects of methyl jasmonate on nitrate uptake, senescence, growth, and VSP accumulation. J Exp Bot. 2002;53(371):1131–41. 10.1093/jexbot/53.371.1131 11971924

[130] Hristova VA, Popova LP. Treatment with methyl jasmonate alleviates the effects of paraquat on photosynthesis in barley plants. Photosynthetica. 2002;40(4):567–74.

[131] Gomez S, Ferrieri RA, Schueller M, Orians CM. Methyl jasmonate elicits rapid changes in carbon and nitrogen dynamics in tomato. New Phytol. 2010;188(3):835–44. 10.1111/j.1469-8137.2010.03414.x . 20723074

[132] Nabity PD, Zavala JA, DeLucia EH. Herbivore induction of jasmonic acid and chemical defences reduce photosynthesis in *Nicotiana attenuata*. J Exp Bot. 2013;64(2):685–94. 10.1093/jxb/ers364 23264519 PMC3542056

[133] Ferrieri AP, Agtuca B, Appel HM, Ferrieri RA, Schultz JC. Temporal changes in allocation and partitioning of new carbon as ^11^C elicited by simulated herbivory suggest that roots shape aboveground responses in *Arabidopsis*. Plant Physiol. 2013;161(2):692–704. 10.1104/pp.112.208868 23370716 PMC3561013

[134] Xiong Y, McCormack M, Li L, Hall Q, Xiang C, Sheen J. Glucose-TOR signalling reprograms the transcriptome and activates meristems. Nature. 2013;496(7444):181–6. 10.1038/nature12030 23542588 PMC4140196

[135] Lemmermeyer S, Lörcher L, Kleunen Mv, Dawson W. Testing the plant growth-defense hypothesis belowground: do faster-growing herbaceous plant species suffer more negative effects from soil biota than slower-growing ones? Am Nat. 2015;186(2):264–71. 10.1086/682005 . 26655154

[136] Fine PVA, Miller ZJ, Mesones I, Irazuzta S, Appel HM, Stevens MHH, et al The growth–defense trade-off and habitat specialization by plants in amazonian forests. Ecology. 2006;87(sp7):S150–S62. 10.1890/0012-9658(2006)87[150:TGTAHS]2.0.CO;2 16922310

