# Supplementary figures and images for: Transcriptomic Analysis of Responses to Imbalanced Carbon: Nitrogen Availabilities in Rice Seedlings
Source: PLoS One. 2016 Nov 7;11(11):e0165732. doi: 10.1371/journal.pone.0165732 (PMC5098742; doi:10.1371/journal.pone.0165732)

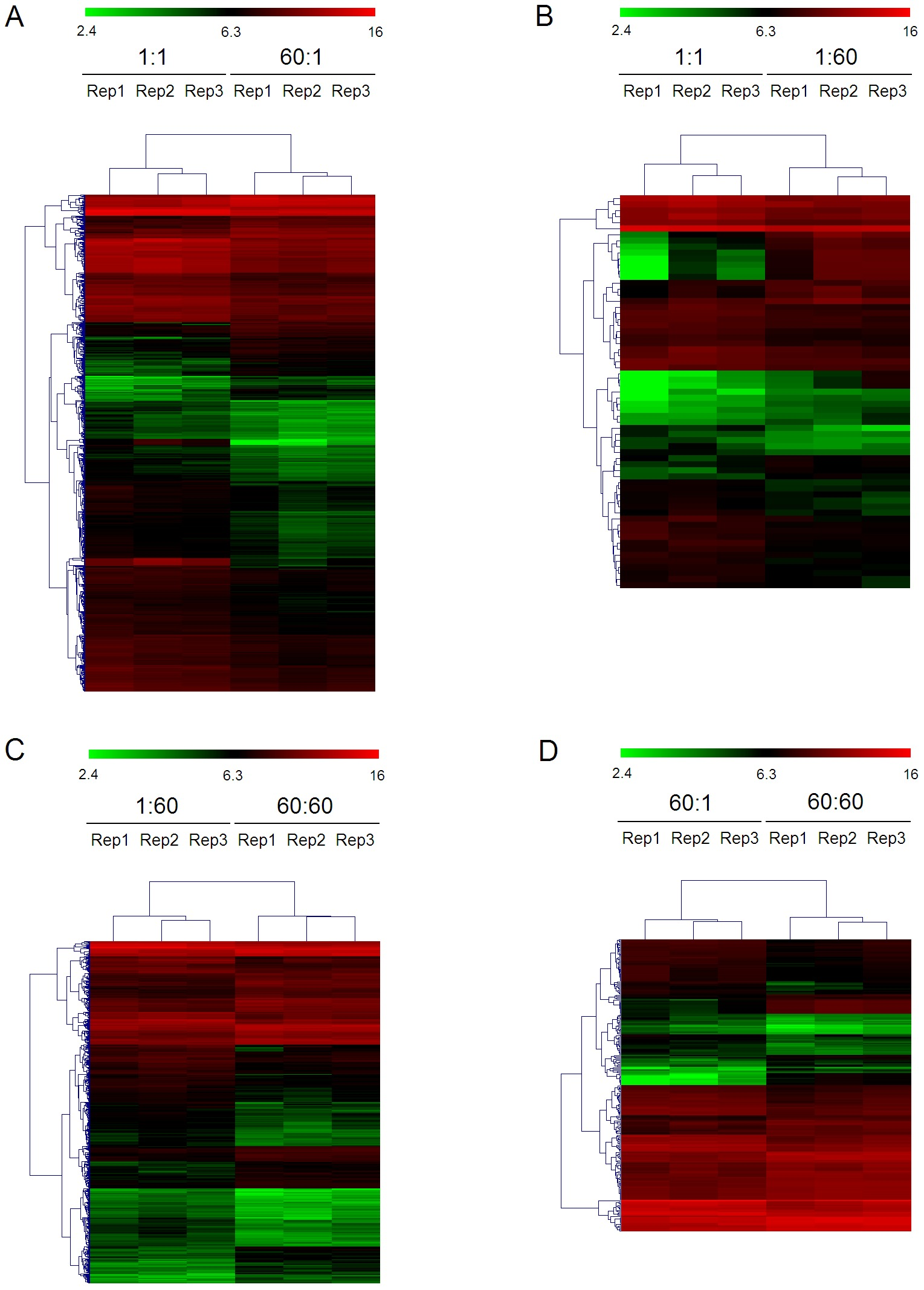

Supplement: S1 Fig — The log2 ratio values of probe sets were used for the analysis with R software. The colored bars represent the value (log2(fold change)) of the transcripts in each bin after CN treatments. Green represents down-regulated probe sets, red represents up-regulated probe sets, and dark indicates no significant difference in gene expression. (A) Hierarchical clustering of rice genes (probe sets) between 1:1 and 60:1; (B) Hierarchical clustering of rice genes (probe sets) between 1:1 and 1:60; (C) Hierarchical clustering of rice genes (probe sets) between 1:60 and 60:60; (D) Hierarchical clustering of rice genes (probe sets) between 60:1 and 60:60. (TIF) [file pone.0165732.s001.tif]
