# Supplementary material for: Transcriptomic Analysis of Responses to Imbalanced Carbon: Nitrogen Availabilities in Rice Seedlings
Source: PLoS One. 2016 Nov 7;11(11):e0165732. doi: 10.1371/journal.pone.0165732 (PMC5098742; doi:10.1371/journal.pone.0165732)
Supplement: S1 Table — (DOCX) [file pone.0165732.s006.docx]

| **Gene** | **RAP locus ID** | **Primer sequences** | **Products’ length (bp)** |
| --- | --- | --- | --- |
| *NR* | *Os08g0468700* | F: CATCATGCTCGCCTACATGCAGAA | 160 |
|  |  | R: CGCGGTTGTCCTTGTAATGGTAGT |  |
| *PEPCase* | *Os08g0366000* | F: ATGAGTGGCCATTCTTCAGGGTCA | 196 |
|  |  | R: ATATCCTTGTGACCGGCAACCTGA |  |
| *GS* | *Os10g0456500* | F: AGGAGCAAAGCGAGGACTGTGAAA | 105 |
|  |  | R: TGTAGCAGTCGCACATCACAAGGA |  |
| *GOGAT* | *Os07g0658400* | F: AAGTCTGGAAGGAAGGGCTTGGAA | 142 |
|  |  | R: ACTGCTAAGAGCGAACTGACAGCA |  |
| *PK* | *Os11g0216000* | F: TGAAGGATGGCAAGCCAATCAAGC | 162 |
|  |  | R: AGATTGTACCATCGGCGCAGAGAA |  |
| *OsCAB2* | *Os01g0720500* | F: TCTCCATGTTCGGGTTCTT | 102 |
|  |  | R: CCCATGCGTTGTTGTTGA |  |
| *OsPERO* | *Os03g0339300* | F: GCGTCGATCTACTGTACCTA | 110 |
|  |  | R: TCACGTTGCGCACTTATAC |  |
| *OsOPR5* | *Os06g0216300* | F: GCACCATTGAACAGGTATGA | 104 |
|  |  | R: GTTCTTGACTCCTCATCAATCT |  |
| *OsAOS2* | *Os03g0225900* | F: AATACGTGTACTGGTCGAATG | 107 |
|  |  | R: GACGAGCAACAGCCTTC |  |
| *OsLOX1B* | *Os08g0509100* | F: GGACAGGAAGCTCAAGAATC | 131 |
|  |  | R: AGTAGATGTCCTCCCATCAG |  |
| *OsCHS* | *Os07g0526400* | F: GGGTGATGCTGGGATTTG | 111 |
|  |  | R: GTTTGCGTTCACTTACCTTTG |  |
